# Confirmation of inhibitingTLR4/MyD88/NF-κB Signalling Pathway by Duhuo Jisheng Decoction on Osteoarthritis: A Network Pharmacology Approach-Integrated Experimental Study

**DOI:** 10.3389/fphar.2021.784822

**Published:** 2022-01-24

**Authors:** Linglong Liu, Limei Xu, Shengjie Wang, Lili Wang, Xiaoning Wang, Huifeng Xu, Xihai Li, Hongzhi Ye

**Affiliations:** ^1^ College of Integrative Medicine, Fujian University of Traditional Chinese Medicine, Fuzhou, China; ^2^ Academy of Integrative Medicine, Fujian University of Traditional Chinese Medicine, Fuzhou, China; ^3^ College of Pharmacy Science, Fujian University of Traditional Chinese Medicine, Fuzhou, China; ^4^ Fujian Key Laboratory of Integrative Medicine on Geriatrics, Fuzhou, China

**Keywords:** network pharmacology approach, complex herbal formulations, molecular targets, osteoarthritis, Duhuo Jisheng decoction, inflammatory

## Abstract

This study was conducted to identify whether the TLR4/MyD88/NF-κB signalling pathway plays a vital role in osteoarthritis (OA) treatment with Duhuo Jisheng Decoction (DHJSD) on the basis of a network pharmacology approach (NPA)-integrated experiment. Two experiments were conducted as follow: NPA for DHJSD using six OA-related gene series and the key pathway was screened out using NPA. NPA identified a vital role for the TLR4/MyD88/NF-κB signalling pathway in OA treatment with DHJSD, the conventional western blot analysis and qPCR confirmed it. Furthermore, changes of miR-146a-5p and miR-34a-5p in the cellular models were recovered by DHJSD administration, which synergistically contributed to OA therapy. The toll-like receptor signalling pathway and the NF-κB signalling pathway were meaningfully enriched by the miRNA-regulated gene pathways. This study identified and confirmed the TLR4/MyD88/NF-κB signalling pathway is an essential inflammatory signalling pathway in the DHJSD underlying OA treatment. The results provide a basis for further evaluation of the regulatory mechanism of the drug’s efficacy in treating OA.

## Introduction

Osteoarthritis (OA) is a progressive, degenerative disease characterised by inflammation-driven cartilage degradation in the elderly (Kwon et al., 2018; Funck-Brentano et al., 2019; Peat and Thomas, 2021). OA resulting in joint chronic pain and disability can lead to remarkable reduction of the quality of life, which poses an increasing socioeconomic and healthcare burden. However, prolonged use of non-steroidal anti-inflammatory drugs for OA treatment may lead to cardiovascular and gastrointestinal side effects (UK NCGC, 2014; Latourte et al., 2020). Pharmacological treatments for OA are aimed at alleviating the clinical symptoms rather than treating the underlying causes. Hence it is in desperate need of more effective and safer therapeutic approaches to reduce chronic pain and functional recovery of joint in patients with OA.

In the field of traditional Chinese medicine (TCM), Chinese herbal medicine has a deep history and special features for OA treatment, and it has been explored to be effective in improving clinical symptoms with few side effects. Duhuo Jisheng Decoction (DHJSD) has been widely used to treat OA in China, and has been confirmed to relieve OA-related symptoms in several clinical trials (Zhang et al., 2016). Studies have shown that DHJSD could alleviate OA by suppressing inflammation and chondrocyte apoptosis (Liu et al., 2018; Liu et al., 2020). Our previous studies shown that DHJSD inhibits chondrocyte apoptosis by affect the mitochondrial-dependent apoptotic pathway (Liu et al., 2014) and suppresses the endoplasmic reticulum stress-mediated apoptosis (Lin et al., 2015). Some clues about the pharmacological mechanism of DHJSD have been provided, indicating that its pharmacological efficacy may through the synergistic actions of the multi-ingredients modulating multi-pathways. Nevertheless, the specific pharmacological mechanisms of DHJSD and their interaction with OA-related targets and pathways is not yet elucidated and require further investigation.

Network pharmacology approach (NPA), a novel and powerful strategy, integrates chemo-informatics, bio-informatics, network biology, network analysis and traditional pharmacology, which conforms to an organic whole or the holistic view of TCM theory and elucidates the active compounds and potential mechanisms of TCM formulas. Classical network pharmacology explores molecular targets from the ingredients of the complex herbal formulation and the target disease, simultaneously and refines the intersections between the formulations and disease to identify the therapeutic molecular targets (Lai et al., 2020). NPA has been beneficial in elucidating the interrelationship of the compounds and construct the compound, compound target, and target disease networks to exploring the possible mechanisms of multi-component and multi-target drugs (Liu et al., 2016). Jian et al. used NPA to investigate the effects of Eucommia ulmoides-Radix Achyranthis Bidentatae (EU-RAB) in OA, showed that TNF, MAPK, PI3K/AKT and IL-17 signalling pathways may be pathway that EU-RAB affects in OA (Jian et al., 2020). Zhu et al. reported that cell cycle, cell apoptosis, immune modulation, inflammation and drug metabolism maybe the pathway that Shaoyao Gancao (SGD) affects in OA by NPA analysis (Zhu et al., 2019). Recently, Zhang et al. found that virus-related, apoptotic, IL-17 and PI3K/Akt signalling pathway are involved in the mechanisms of Radix Achyranthis Bidentatae in treating acute OA using NPA (Zhang et al., 2020), Huang et al. used NPA and found that Simiao may exert its effect on multi-pathway such as IL-17 signalling pathway, HIF-1 signalling pathway, Toll-like receptor signalling pathway and TNF signalling pathway (Huang et al., 2020). In our previous study, we also used NPA and OA in a cellular model and found that the NF-κB signalling pathway was the important inflammatory signalling pathway involved in the mechanism of Bushen Zhuangjin Decoction (BZD) in treating OA (Xu et al., 2021). These studies had focused on investigating the potential molecular targets and mechanisms of a certain herbal formulation using NPA. Therefore, using NPA-integrated experimental to acquire the bioactive components, targets and the underlying mechanisms of DHJSD in treating OA. The following two experiments were designed: 1) network pharmacology for DHJSD and 2) variation of the pathway detected from network pharmacology and confirming the key signalling pathway of DHJSD on a lipopolysaccharide (LPS) induced OA cellular model. This study provides a newish approach to understand the theoretical basis of DHJSD in treating OA.

## Methods and Materials

### Preliminary Exploration of the Bioactive Components of DHJSD

#### Preparation of DHJSD

DHJSD is formed from 15 Chinese medical plants, including Duhuo (DH) [Angelica biserrata (Shan et Yuan) Yuan et Shan], Sang Ji Sheng (SJS) [Taxillus chinensis (DC.) Danser], Qin Jian (QJ) [Gentiana Macrophylla Pall.], Fang Feng (FF) [Saposhnikovia divaricata (Turcz.) Schischk.], Xi Xin (XX) [Manchurian Wildginger], Chuan Xiong (CX) [Szechuan Lovage Rhizome], Dang Gui (DG) [Angelica sinensis (Oliv.) Diels.], Shu Di Huang (SDH) [Rehmanniaglutinosa (Gaertn.) DC.], Bai Shao (BS) [Paeonia lactiflora Pall], Rou Gui (RG) [Cortex Cinnamomi Cassiae], Fu Ling (FL) [Smilax glabra Roxb], Du Zhong (DZ) [Eucommia ulmoides Oliv], Niu Xi (NX) [Radix Achyranthis Bidentatae], Dang Shen (DS) [Root of Pilose Asiabell], Gan Cao (GC) [Glycyrrhiza uralensis Fisch.], were obtained from the Third People’s Hospital affiliated to the Fujian University of TCM (Fuzhou, China). Regarding the ratio of DHJSD (Supplementary Table S1), 90 g of herbal powder was soaked in 720 ml distilled water and boiled three times for 2 h, and the extracts were filtered. The filtrate was completely evaporated on a rotary evaporator (RE-2000; Shanghai Yarong Biochemistry Instrument Factory, Shanghai, China), dried in vacuum drying oven until it was constant weight, and then stored at −20 °C (DZF-300; Shanghai Yiheng Scientific Instrument Co., Shanghai, China). The DHJSD solid dry matter was dissolved in Dulbecco’s modified Eagle’s medium (HyClone, Logan, UT, United States) and then filtered through a 0.22 µm filter. When used, was prepared as necessary. The extracts of DHJSD were detected by high performance liquid chromatography (HPLC) fingerprint on an Agilent 1200 HPLC system (Agilent, Santa Clara, CA,United States) using an Agilent 5 TC-C_18_ (250 × 4.6 mm). The conditions for the analysis were acetonitrile (A) 0.2% phosphoric acid water (B) as a mobile phase, a detection wavelength at 230 nm for paeoniflorin (peak 1) (purity 98%), ligustrazine hydrochloride (peak 2) (purity 98%) and osthole (peak 3) (purity 98%) (China Institute of Food and Drug Test, Beijing, China) (Supplementary Figure S1), with flow rate of 0.8 ml/min under column temperature.

#### Screening of the Bioactive Components of DHJSD

All DHJSD components were explored using the TCM systems pharmacology database and analysis platform (TCMSP, https://tcmspw.com/tcmsp.php). The chemical composition of TCM was screened based on oral bioavailability (OB ≥ 30%) and drug-likeness (DL ≥ 0.18). The OB value describe the relative amount drug which was absorbed into the systemic blood circulation, through the extravascular route. DL means the degree of similarity between bioactive component and a known drug, and the class of compounds with the potential for drug development. Moreover, 206 eligible DHJSD compounds were obtained from the TCM systems pharmacology database, and considered as candidate compounds (Table 1), including 9 for DH, 2 for QJ, 2 for SJS, 8 for XX, 0 for RG, 18 for FF, 15 for FL, 2 for SDH, 20 for NX, 21 for DS, 28 for DZ, 7 for CX, 2 for DG, 13 for BS and 92 for GC.

**TABLE 1:** The final selected compounds of DuHuo JiSheng Decoction for analysis.

ID	Name	OB	DL	Herb
MOL003608	O-Acetylcolumbianetin	60.04	0.26	DH
MOL004777	Angelol D	34.85	0.34	DH
MOL004778	[(1R,2R)-2,3-dihydroxy-1-(7-methoxy-2-oxochromen-6-yl)-3-methylbutyl] (Z)-2-methylbut-2-enoate	46.03	0.34	DH
MOL004780	Angelicone	30.99	0.19	DH
MOL004782	[(1R,2R)-2,3-dihydroxy-1-(7-methoxy-2-oxochromen-6-yl)-3-methylbutyl] 3-methylbutanoate	45.19	0.34	DH
MOL004792	Nodakenin	57.12	0.69	DH
MOL000011	(2R,3R)-3-(4-hydroxy-3-methoxy-phenyl)-5-methoxy-2-methylol-2,3-dihydropyrano[5,6-h][1,4]benzodioxin-9-one	68.83	0.66	FF
MOL011730	11-hydroxy-sec-o-beta-d-glucosylhamaudol_qt	50.24	0.27	FF
MOL011732	Anomalin	59.65	0.66	FF
MOL011737	Divaricatacid	87	0.32	FF
MOL011740	Divaricatol	31.65	0.38	FF
MOL011747	Ledebouriellol	32.05	0.51	FF
MOL011749	Phelloptorin	43.39	0.28	FF
MOL011753	5-O-Methylvisamminol	37.99	0.25	FF
MOL002644	Phellopterin	40.19	0.28	FF
MOL003588	Prangenidin	36.31	0.22	FF
MOL013077	Decursin	39.27	0.38	FF
MOL012140	4,9-dimethoxy-1-vinyl-$b-carboline	65.3	0.19	XX
MOL012141	Caribine	37.06	0.83	XX
MOL001460	Cryptopin	78.74	0.72	XX
MOL001558	Sesamin	56.55	0.83	XX
MOL002501	[(1S)-3-[(E)-but-2-enyl]-2-methyl-4-oxo-1-cyclopent-2-enyl] (1R,3R)-3-[(E)-3-methoxy-2-methyl-3-oxoprop-1-enyl]-2,2-dimethylcyclopropane-1-carboxylate	62.52	0.31	XX
MOL002962	(3S)-7-hydroxy-3-(2,3,4-trimethoxyphenyl)chroman-4-one	48.23	0.33	XX
MOL009849	ZINC05223929	31.57	0.83	XX
MOL002135	Myricanone	40.6	0.51	CX
MOL002151	Senkyunone	47.66	0.24	CX
MOL002157	Wallichilide	42.31	0.71	CX
MOL000433	FA	68.96	0.71	CX
MOL001918	Paeoniflorgenone	87.59	0.37	BS
MOL001925	Paeoniflorin_qt	68.18	0.4	BS
MOL001928	Albiflorin_qt	66.64	0.33	BS
MOL001910	11alpha,12alpha-epoxy-3beta-23-dihydroxy-30-norolean-20-en-28,12beta-olide	64.77	0.38	BS
MOL000492	(+)-catechin	54.83	0.24	BS
MOL001924	Paeoniflorin	53.87	0.79	BS
MOL001921	Lactiflorin	49.12	0.8	BS
MOL001919	(3S,5R,8R,9R,10S,14S)-3,17-dihydroxy-4,4,8,10,14-pentamethyl-2,3,5,6,7,9-hexahydro-1H-cyclopenta [a]phenanthrene-15,16-dione	43.56	0.53	BS
MOL001930	Benzoyl paeoniflorin	31.27	0.75	BS
MOL000273	(2R)-2-[(3S,5R,10S,13R,14R,16R,17R)-3,16-dihydroxy-4,4,10,13,14-pentamethyl-2,3,5,6,12,15,16,17-octahydro-1H-cyclopenta [a]phenanthren-17-yl]-6-methylhept-5-enoic acid	30.93	0.81	FL
MOL000275	Trametenolic acid	38.71	0.8	FL
MOL000276	7,9 (11)-dehydropachymic acid	35.11	0.81	FL
MOL000279	Cerevisterol	37.96	0.77	FL
MOL000280	(2R)-2-[(3S,5R,10S,13R,14R,16R,17R)-3,16-dihydroxy-4,4,10,13,14-pentamethyl-2,3,5,6,12,15,16,17-octahydro-1H-cyclopenta [a]phenanthren-17-yl]-5-isopropyl-hex-5-enoic acid	31.07	0.82	FL
MOL000282	Ergosta-7,22E-dien-3beta-ol	43.51	0.72	FL
MOL000283	Ergosterol peroxide	40.36	0.81	FL
MOL000285	(2R)-2-[(5R,10S,13R,14R,16R,17R)-16-hydroxy-3-keto-4,4,10,13,14-pentamethyl-1,2,5,6,12,15,16,17-octahydrocyclopenta [a]phenanthren-17-yl]-5-isopropyl-hex-5-enoic acid	38.26	0.82	FL
MOL000287	3beta-Hydroxy-24-methylene-8-lanostene-21-oic acid	38.7	0.81	FL
MOL000289	Pachymic acid	33.63	0.81	FL
MOL000290	Poricoic acid A	30.61	0.76	FL
MOL000291	Poricoic acid B	30.52	0.75	FL
MOL000292	Poricoic acid C	38.15	0.75	FL
MOL000296	Hederagenin	36.91	0.75	FL
MOL000300	Dehydroeburicoic acid	44.17	0.83	FL
MOL002058	40957-99-1	57.2	0.62	DZ
MOL004367	Olivil	62.23	0.41	DZ
MOL000443	Erythraline	49.18	0.55	DZ
MOL005922	Acanthoside B	43.35	0.77	DZ
MOL006709	AIDS214634	92.43	0.55	DZ
MOL000073	Ent-Epicatechin	48.96	0.24	DZ
MOL007563	Yangambin	57.53	0.81	DZ
MOL009007	Eucommin A	30.51	0.85	DZ
MOL009009	(+)-medioresinol	87.19	0.62	DZ
MOL009015	(-)-Tabernemontanine	58.67	0.61	DZ
MOL009027	Cyclopamine	55.42	0.82	DZ
MOL009029	Dehydrodiconiferyl alcohol 4,gamma'-di-O-beta-D-glucopyanoside_qt	51.44	0.4	DZ
MOL009030	Dehydrodieugenol	30.1	0.24	DZ
MOL009031	Cinchonan-9-al, 6′-methoxy- (9R)-	68.22	0.4	DZ
MOL009038	GBGB	45.58	0.83	DZ
MOL009042	Helenalin	77.01	0.19	DZ
MOL009047	(+)-Eudesmin	33.29	0.62	DZ
MOL009053	4-[(2S,3R)-5-[(E)-3-hydroxyprop-1-enyl]-7-methoxy-3-methylol-2,3-dihydrobenzofuran-2-yl]-2-methoxy-phenol	50.76	0.39	DZ
MOL009055	Hirsutin_qt	49.81	0.37	DZ
MOL009057	Liriodendrin_qt	53.14	0.8	DZ
MOL002773	Beta-carotene	37.18	0.58	DZ
MOL008240	(E)-3-[4-[(1R,2R)-2-hydroxy-2-(4-hydroxy-3-methoxy-phenyl)-1-methylol-ethoxy]-3-methoxy-phenyl]acrolein	56.32	0.36	DZ
MOL011604	Syringetin	36.82	0.37	DZ
MOL012461	28-norolean-17-en-3-ol	35.93	0.78	NX
MOL012505	Bidentatoside, ii_qt	31.76	0.59	NX
MOL012542	β-ecdysterone	44.23	0.82	NX
MOL001454	Berberine	36.86	0.78	NX
MOL001458	Coptisine	30.67	0.86	NX
MOL002643	Delta 7-stigmastenol	37.42	0.75	NX
MOL002714	Baicalein	33.52	0.21	NX
MOL002776	Baicalin	40.12	0.75	NX
MOL002897	Epiberberine	43.09	0.78	NX
MOL003847	Inophyllum E	38.81	0.85	NX
MOL000785	Palmatine	64.6	0.65	NX
MOL000085	Beta-daucosterol_qt	36.91	0.75	NX
MOL012537	Spinoside A	41.75	0.4	NX
MOL008406	Spinoside A	39.97	0.4	DS
MOL002879	Diop	43.59	0.39	DS
MOL003036	ZINC03978781	43.83	0.76	DS
MOL004492	Chrysanthemaxanthin	38.72	0.58	DS
MOL005321	Frutinone A	65.9	0.34	DS
MOL000006	Luteolin	36.16	0.25	DS
MOL006554	Taraxerol	38.4	0.77	DS
MOL006774	Stigmast-7-enol	37.42	0.75	DS
MOL008391	5alpha-Stigmastan-3,6-dione	33.12	0.79	DS
MOL008393	7-(beta-Xylosyl)cephalomannine_qt	38.33	0.29	DS
MOL008397	Daturilin	50.37	0.77	DS
MOL008400	Glycitein	50.48	0.24	DS
MOL008407	(8S,9S,10R,13R,14S,17R)-17-[(E,2R,5S)-5-ethyl-6-methylhept-3-en-2-yl]-10,13-dimethyl-1,2,4,7,8,9,11,12,14,15,16,17-dodecahydrocyclopenta [a]phenanthren-3-one	45.4	0.76	DS
MOL008411	11-Hydroxyrankinidine	40	0.66	DS
MOL001484	Inermine	75.18	0.54	GC
MOL001792	DFV	32.76	0.18	GC
MOL002311	Glycyrol	90.78	0.67	GC
MOL000239	Jaranol	50.83	0.29	GC
MOL002565	Medicarpin	49.22	0.34	GC
MOL000354	Isorhamnetin	49.6	0.31	GC
MOL003656	Lupiwighteone	51.64	0.37	GC
MOL000392	Formononetin	69.67	0.21	GC
MOL000417	Calycosin	47.75	0.24	GC
MOL004328	Naringenin	59.29	0.21	GC
MOL004805	(2S)-2-[4-hydroxy-3-(3-methylbut-2-enyl)phenyl]-8,8-dimethyl-2,3-dihydropyrano [2,3-f]chromen-4-one	31.79	0.72	GC
MOL004806	Euchrenone	30.29	0.57	GC
MOL004808	Glyasperin B	65.22	0.44	GC
MOL004810	Glyasperin F	75.84	0.54	GC
MOL004811	Glyasperin C	45.56	0.4	GC
MOL004814	Isotrifoliol	31.94	0.42	GC
MOL004815	(E)-1-(2,4-dihydroxyphenyl)-3-(2,2-dimethylchromen-6-yl)prop-2-en-1-one	39.62	0.35	GC
MOL004820	Kanzonols W	50.48	0.52	GC
MOL004824	(2S)-6-(2,4-dihydroxyphenyl)-2-(2-hydroxypropan-2-yl)-4-methoxy-2,3-dihydrofuro [3,2-g]chromen-7-one	60.25	0.63	GC
MOL004827	Semilicoisoflavone B	48.78	0.55	GC
MOL004828	Glepidotin A	44.72	0.35	GC
MOL004829	Glepidotin B	64.46	0.34	GC
MOL004833	Phaseolinisoflavan	32.01	0.45	GC
MOL004835	Glypallichalcone	61.6	0.19	GC
MOL004838	8-(6-hydroxy-2-benzofuranyl)-2,2-dimethyl-5-chromenol	58.44	0.38	GC
MOL004841	Licochalcone B	76.76	0.19	GC
MOL004848	Licochalcone G	49.25	0.32	GC
MOL004849	3-(2,4-dihydroxyphenyl)-8-(1,1-dimethylprop-2-enyl)-7-hydroxy-5-methoxy-coumarin	59.62	0.43	GC
MOL004855	Licoricone	63.58	0.47	GC
MOL004856	Gancaonin A	51.08	0.4	GC
MOL004857	Gancaonin B	48.79	0.45	GC
MOL004860	Licorice glycoside E	32.89	0.27	GC
MOL004863	3-(3,4-dihydroxyphenyl)-5,7-dihydroxy-8-(3-methylbut-2-enyl)chromone	66.37	0.41	GC
MOL004864	5,7-dihydroxy-3-(4-methoxyphenyl)-8-(3-methylbut-2-enyl)chromone	30.49	0.41	GC
MOL004866	2-(3,4-dihydroxyphenyl)-5,7-dihydroxy-6-(3-methylbut-2-enyl)chromone	44.15	0.41	GC
MOL004879	Glycyrin	52.61	0.47	GC
MOL004882	Licocoumarone	33.21	0.36	GC
MOL004883	Licoisoflavone	41.61	0.42	GC
MOL004884	Licoisoflavone B	38.93	0.55	GC
MOL004885	Licoisoflavanone	52.47	0.54	GC
MOL004891	Shinpterocarpin	80.3	0.73	GC
MOL004898	(E)-3-[3,4-dihydroxy-5-(3-methylbut-2-enyl)phenyl]-1-(2,4-dihydroxyphenyl)prop-2-en-1-one	46.27	0.31	GC
MOL004903	Liquiritin	65.69	0.74	GC
MOL004904	Licopyranocoumarin	80.36	0.65	GC
MOL004905	3,22-Dihydroxy-11-oxo-delta (12)-oleanene-27-alpha-methoxycarbonyl-29-oic acid	34.32	0.55	GC
MOL004907	Glyzaglabrin	61.07	0.35	GC
MOL004908	Glabridin	53.25	0.47	GC
MOL004910	Glabranin	52.9	0.31	GC
MOL004911	Glabrene	46.27	0.44	GC
MOL004912	Glabrone	52.51	0.5	GC
MOL004913	1,3-dihydroxy-9-methoxy-6-benzofurano [3,2-c]chromenone	48.14	0.43	GC
MOL004914	1,3-dihydroxy-8,9-dimethoxy-6-benzofurano [3,2-c]chromenone	62.9	0.53	GC
MOL004915	Eurycarpin A	43.28	0.37	GC
MOL004917	Glycyroside	37.25	0.79	GC
MOL004924	(-)-Medicocarpin	40.99	0.95	GC
MOL004935	Sigmoidin-B	34.88	0.41	GC
MOL004941	(2R)-7-hydroxy-2-(4-hydroxyphenyl)chroman-4-one	71.12	0.18	GC
MOL004945	(2S)-7-hydroxy-2-(4-hydroxyphenyl)-8-(3-methylbut-2-enyl)chroman-4-one	36.57	0.32	GC
MOL004948	Isoglycyrol	44.7	0.84	GC
MOL004949	Isolicoflavonol	45.17	0.42	GC
MOL004957	HMO	38.37	0.21	GC
MOL004959	1-Methoxyphaseollidin	69.98	0.64	GC
MOL004961	Quercetin der	46.45	0.33	GC
MOL004966	3′-Hydroxy-4′-O-Methylglabridin	43.71	0.57	GC
MOL000497	Licochalcone a	40.79	0.29	GC
MOL004974	3′-Methoxyglabridin	46.16	0.57	GC
MOL004978	2-[(3R)-8,8-dimethyl-3,4-dihydro-2H-pyrano [6,5-f]chromen-3-yl]-5-methoxyphenol	36.21	0.52	GC
MOL004980	Inflacoumarin A	39.71	0.33	GC
MOL004985	Icos-5-enoic acid	30.7	0.2	GC
MOL004988	Kanzonol F	32.47	0.89	GC
MOL004989	6-prenylated eriodictyol	39.22	0.41	GC
MOL004990	7,2′,4′-trihydroxy-5-methoxy-3-arylcoumarin	83.71	0.27	GC
MOL004991	7-Acetoxy-2-methylisoflavone	38.92	0.26	GC
MOL004993	8-prenylated eriodictyol	53.79	0.4	GC
MOL004996	Gadelaidic acid	30.7	0.2	GC
MOL000500	Vestitol	74.66	0.21	GC
MOL005000	Gancaonin G	60.44	0.39	GC
MOL005001	Gancaonin H	50.1	0.78	GC
MOL005003	Licoagrocarpin	58.81	0.58	GC
MOL005007	Glyasperins M	72.67	0.59	GC
MOL005008	Glycyrrhiza flavonol A	41.28	0.6	GC
MOL005012	Licoagroisoflavone	57.28	0.49	GC
MOL005013	18α-hydroxyglycyrrhetic acid	41.16	0.71	GC
MOL005016	Odoratin	49.95	0.3	GC
MOL005017	Phaseol	78.77	0.58	GC
MOL005018	Xambioona	54.85	0.87	GC
MOL005020	Dehydroglyasperins C	53.82	0.37	GC
MOL000098	Quercetin	46.43	0.28	DZ/SJS
MOL000098	Quercetin	46.43	0.28	GC/NX
MOL000173	Wogonin	30.68	0.23	FF/NX
MOL000211	Mairin	55.38	0.78	BS/DZ/GC
MOL000358	Beta-sitosterol	36.91	0.75	DH/QJ/FF/DG/BS/DZ/NX
MOL000359	Sitosterol	36.91	0.75	SJS/QJ/FF/CX/SDH/BS/GC
MOL000422	Kaempferol	41.88	0.24	XX/BS/DZ/NX/GC
MOL000449	Stigmasterol	43.83	0.76	DG/SDH/NX/DS
MOL001006	poriferasta-7,22E-dien-3beta-ol	42.98	0.76	NX/DS
MOL001494	Mandenol	42	0.19	FF/CX
MOL001941	Ammidin	34.55	0.22	DH/FF
MOL001942	Isoimperatorin	45.46	0.23	DH/FF
MOL002140	Perlolyrine	65.95	0.27	CX/DS
MOL003896	7-Methoxy-2-methyl isoflavone	42.56	0.2	DS/GC
MOL004355	Spinasterol	42.98	0.76	NX/DS
MOL007059	3-beta-Hydroxymethyllenetanshiquinone	32.16	0.41	DZ/DS
MOL007514	Methyl icosa-11,14-dienoate	39.67	0.23	FF/DS

OB, oral bioavailability; DL, drug-likeness; DH, Du Huo; SJS, Sang Ji Sheng; QJ, Qin Jiao; FF, Fang Feng; XX, Xi Xin; CX, Chuan Xiong; DG, Dang Gui; SDH, Shu Di Huanga; BS, Bai Shao; RG, Rou Gui; FL, Fu Ling; DZ, Du Zhong; NX, Niu Xi; DS, Dang Shen; GC, Gan Cao.

#### Animals

Sprague Dawley rats aged 4 weeks (BW: 90–120g, n = 24) were purchased from Shanghai Slack Laboratory Animal Co. (Shanghai, China). Rats were raised in the SPF-class housing of laboratory at 60% humidity, 23°C room temperature and 12 h rhythm (8:00 a.m.-8:00 p.m.) with food and water freely available. The animals were carefully treated according to the National Institutes of Health Guidelines for the Care and Use of Laboratory Animals, and the Animal Care and Use Committee of the Fujian University of TCM play a major role in approved and supervised the experimental (approval number: 2020015).

#### Preparation of Chondrocytes and LPS-Exposed Model

Chondrocytes were harvested and produced to an LPS-exposed cellular model as done in our previous studies (Liang et al., 2019; Li et al., 2020a; Li et al., 2020b). Briefly, chondrocytes were harvested from the knee joints of four Sprague Dawley rats at a time, six times in total. Cells were identified with collagen II antibody by immunohistochemistry. The cellular model was built by exposed Chondrocytes under10 ng/mL of LPS (Sigma-Aldrich, United States) for 8 h.

### Identification of the Key Pathway Concerning DHJSD in Treating OA by NPA

#### Predicting the Molecular Targets of OA

Genetic date on patients with OA acquisition from the Gene Expression Omnibus database (GEO, https://www.ncbi.nlm.nih.Gov/geo/). The series GSE46750 was an analysis of synovial cells from OA patients’ local tissue of inflammatory (I) or normal/reactive (N/R). Differential expression patterns were identified in two regions of the synovium in 12 patients undergoing total knee arthroplasty. The series GSE82107 was an analysis of synovial biopsies that were obtained from the synovium of patients with OA to identify genes upregulated during OA. A total of 10 microarrays of end-stage OA synovial biopsies were compared with 7 microarrays of synovial biopsies obtained from individuals without a joint disease. The series GSE51588 was isolated from total RNA from the regions of interest from OA (n = 20) and non-OA (n = 5) patients, samples were got from knee lateral (LT) and medial tibial (MT) plateaus. This profile was obtained by performing whole-genome microarray profiling of the human osteoarthritic subchondral bone. The series GSE117999 was an analysis of cartilage tissues obtained from 12 OA patients 12 normal patients. The series GSE32317 characterised analyse the synovial membranes from early-stage or end-stage OA patients. A total of 10 samples of early-stage knee OA synovial membranes from the patients undergoing arthroscopic procedures for degenerative meniscal tears and 9 samples of end-stage knee OA synovial membranes. The series GSE29746 was used to identify difference expression gene between human synovial fibroblasts (SF) from OA synovial tissues and normal SF from healthy individuals (HSF). Synovial fibroblasts were obtained from 11 sex-matched and age-matched adult healthy donors (HSF) and 11 sex-matched and age-matched patients with OA (OASF). The raw file was processed by a robust multi-array average algorithm with normalisation of matrix data, and then filtered using the Limma package to analyse twice. The genes with *p* < 0.05 and |log 2 (fold change)| > 0.263 were screened as significantly differentially expressed genes. The analysis data obtained from the two series GSE46750 and GSE82107 were integrated as series 1, the analysis data obtained from the two series GSE51588 and GSE117999 were integrated as series 2 and the analysis data obtained from the two series GSE32317 and GSE29746 were integrated as series 3.

#### Construction of the Active Component-Target Network of DHJSD

The compound target network of DHJSD was constructed and visualised by the Cytoscape 3.7.2 software. Protein–protein interaction (PPI) data that contain many database including the Database of Interacting Proteins (DIP™), Biological General Repository for Interaction Datasets, Human Protein Reference Database, IntAct Molecular Interaction Database (IntAct), Molecular Interaction database (MINT) and the Biomolecular Interaction Network Database. The Cytoscape3.7.2 software provides a visual for the PPI networks for DHJSD putative targets and OA-related targets.

#### Construction of PPI Networks and Screening of Key Targets

In this study, the Biogenet plugin was used to construct the PPI networks for DHJSD and OA targets, and Cytoscape was further to intersection of the two networks, screened the direct or indirect target regulatory network for DHJSD in OA treatment. Key genes were identified based on the network topology analysis plugin CytoNCA and filter out by degree centrality (DC), betweenness centrality (BC) and closeness centrality (CC) and explored for the core genes for DHJSD activity against OA.

#### Gene Ontology and KEGG Analysis

The Gene Ontology (GO) database (http://geneontology.org/) includes biological process (BP), molecular function (MF) and cellular component (CC) data, and it was used to identify the biological mechanisms of a number of large genomes. The top 20 functional categories of each category were selected (FDR< 0.05). The KEGG database (https://www.kegg.jp/) was used to explore possible function and biological correlation with candidate target genes. The Cluster Profiler R package was used to visualise the result of GO and KEGG pathway enrichment. Pathways with an FDR of <0.05 were selected for the next step analysis. The gene pathway network analysis was used to determine significantly regulated pathways. To screen key target genes involved in DHJSD activity, we constructed the gene pathway network.

#### Component-Target Molecular Docking

The 3D structure of the key target genes was downloaded from the RSCB PDB database (https://www.rcsb.org/), the Auto Dock Tools 1.5.6 software was used to remove water and ligands and add hydrogen and stored as PDBQT file. The 2D structures of the top nine compounds of DHJSD were downloaded from the PubChem database (https://pubchem.ncbi.nlm.nih.gov/), processed, transformed into PDB format by Chem3D and stored as PDBQT file. The key target genes were provided as receptors, and the active compounds were provided as ligands. Finally, Autodock vina 1.1.2 was used to dock compounds with key target genes and protein.

#### Venn Analysis

The key target genes of intersections among series 1, series 2 and series 3 were screened by Venn analysis. The results of the key target genes of DHJSD, interactive PPI network topological analysis were submitted for Venn analysis. KEGG was performed to further analysis the genes dropped in the intersections of the target genes of DHJSD along with PPI network topological analysis. Finally, the key pathways concerning DHJSD in OA treatment were identified.

### Verification of the Involvement of Targeted Pathways

#### Verification of the Efficacy of DHJSD on OA Related miRNA and Regulatory Pathway Prediction

Total RNA was extracted from chondrocytes using the TRIZOL reagent (Life, United States) and reverse transcribed into cDNA using RevertAid First Strand cDNA Synthesis kit (PrimeScript™ RT reagent kit). The mRNA expressions of miR-146a-5p and miR-34a-5p were detected using the ABI 7500 Fast Real-time PCR system. The primers used for amplification were as follows: miR-146a-5p (sequence UGA GAA CUG AAU UCC AUG GGU U, forward TGA GAA CTG AAT TCC ATG GGT T) and miR-34a-5p (sequence UGG CAG UGU CUU AGC UGG UUG U, forward TGG CAG TGT CTUT AGC TGG TTG T). The DIANA Tools (http://www.microrna.gr) was used to predict miRNA target mRNAs (Vlachos and Hatzigeorgiou, 2017), and we also analysed the miRNA target mRNA regulatory pathways.

#### Verification of the Efficacy of DHJSD by Western Blotting

It is found that the protein expression of MMP-9 in chondrocytes (LPS 10 ng/ml) was significantly reduced after culture under 300 μg/ml of DHJSD for 8 h in our previous research (Xu et al., 2019). Therefore, we selected 300 μg/ml of DHJSD and a duration of 8 h as the experimental conditions. According to NPA results, we also performed standard western blot analysis was used to confirm the involvement of the targeted pathway. We confirmed the involvement of the NF-κB signalling pathway and the toll-like receptor signalling pathway (Please refer to the Results and Discussion section). Chondrocytes were subjected to four treatment group, namely, control, model (LPS 10 ng/ml), DHJSD (DHJSD 300 μg/ml + LPS 10 ng/ml) and PDTC (an inhibitor of the NF-κB signalling pathway) (PDTC 10 µM + LPS 10 ng/ml) or TAK 242 (an inhibitor of the toll-like receptor four signalling pathway) (TAK 242 1 µM + LPS 10 ng/ml) for 8 h. Cell were lysed immediately in lysis buffer (Beyotime Institute of Biotechnology, Haimen, China) for 30 min on ice, and total proteins were collected and quantified by the bicinchoninic acid assay. Protein sample (20 µg) was run on a 10% SDS-PAGE gel and then transferred protein onto polyvinylidene fluoride membranes (Sigma-Aldrich, United States). Then, 5% non-fat milk was used to block the membranes before incubation with primary antibodies against NF-κB p65 (ab16502, abcam, United States), IKK-β (ab124957, abcam, United States), TLR4 (ab22048, abcam, United States), MyD88 (ab 2064, abcam, United States), TRAF6 (ab40675, abcam, United States), IKK-α (ab32041, abcam, United States), IKB-α (ab32518, abcam, United States), *p*-IKB-α (ab2859, abcam, United States), ADAMTS-4 (ab185722, abcam, United States), ADAMTS-5 (ab41037, abcam, United States), MMP-3 (ab52915, abcam, United States), MMP-13 (ab39012, abcam, United States), MMP-14 (ab51074, abcam, United States), collagen Ⅱ (ab34712, abcam, United States) and *β*-actin (ab8226, Cambridge, UK) at 4°C overnight. Goat anti-rabbit horseradish peroxidase-conjugated secondary antibody IgG (bs-0295G-HRP, Bioss, China) or goat anti-mouse horseradish peroxidase-conjugated secondary antibody IgG (bs-0296G-HRP, Bioss, China) was added to the membranes at 30°C column temperature. The enhanced chemiluminescence method was used to visualize the immunocomplexes. Protein bands were quantified by densitometry scanning (X-ray Spectroscopy System, cat. no. 170-8070; Bio-Rad). The Blots was analyzed using the Image Lab software, *β*-actin used as the control.

#### Quantitative Real-Time PCR (qPCR) Assay

The mRNA expression levels of TLR4, TRAF-6, MyD88, NF-κB p65, IKK-α, IKK-β, MMP-3, MMP-13, MMP-14, IL-1β and TNF-α were detected by using the ABI 7500 Fast Real-time PCR system. The primers used for amplification were as follows:TLR4 (forward 5′-CCG CTC TGG CAT CAT CTT CA-3′, reverse 5′-CTC CCA CTC GAG GTA GGT GT-3′); TRAF-6 (forward 5′-TGG TCA TTC ATA GCC CTG GAT-3′, reverse 5′-AGG ATC GTG AGG CGT ATT GTA-3′); MyD88 (forward 5′-CGG AGG AGA TGG GTT TCG AG-3′, reverse 5′-CCA GGC ATC CAA CAA ACT GC-3′); NF-κB p65 (forward 5′-AGA GAA GCA CAG ATA CCA CTA AGA-3′, reverse 5′-GTT CAG CCT CAT AGA AGC CAT C-3′); IKK-α (forward 5′-TGG AGT GAG AGG CTG TGA TAG-3′, reverse 5′-TTG AGT TGC TGT GAT GCT GAG-3′); IKK-β (forward 5′-CAT CAA GCA ATG CCG ACA GG-3′, reverse 5′-CAT TGG GTG CCA AGT TCT GC-3′); MMP-3 (forward 5′-ATG ATG ATG AAC GAT GGA CAG ATG-3′, reverse 5′-TGC TAC ACA TTG GTA AGG TCT CAG-3′); MMP-13 (forward 5′-GGA GTA ATC GCA TTG TGA GAG TC-3′, reverse 5′-GGT TCC AGC CAC GCA TAG TC-3′); MMP-14 (forward 5′-GCA CTT CGT GTT GCC TGA TGA C-3′, reverse 5′-GCC AGA ACC ATC GCT CCT TGA-3′); IL-1β (forward 5′-GCA CAG TTC CCC AAC TGG TA-3′, reverse 5′-AAG ACA CGG GTT CCA TGG TG-3′); TNF-α (forward 5′-ACT GAA CTT CGG GGT GAT CG-3′, reverse 5′-GCT TGG TGG TTT GCT ACG AC-3′). GAPDH was used as an internal control.

### Statistical Analysis

All statistical data were analysed by SPSS 20.0 software (SPSS Inc., Chicago, IL, United States) and expressed as mean ± standard deviation (SD). Data obtained from three independent experiments were analysed by two-way analysis of variance, through Bonferroni’s post-hoc correction for multiple comparisons. *P* < 0.05 was considered to indicate a significant difference.

## Results

### Screening of the Key Pathway of DHJSD Using NPA

#### Compound Target Network Analysis

**FIGURE 1:**
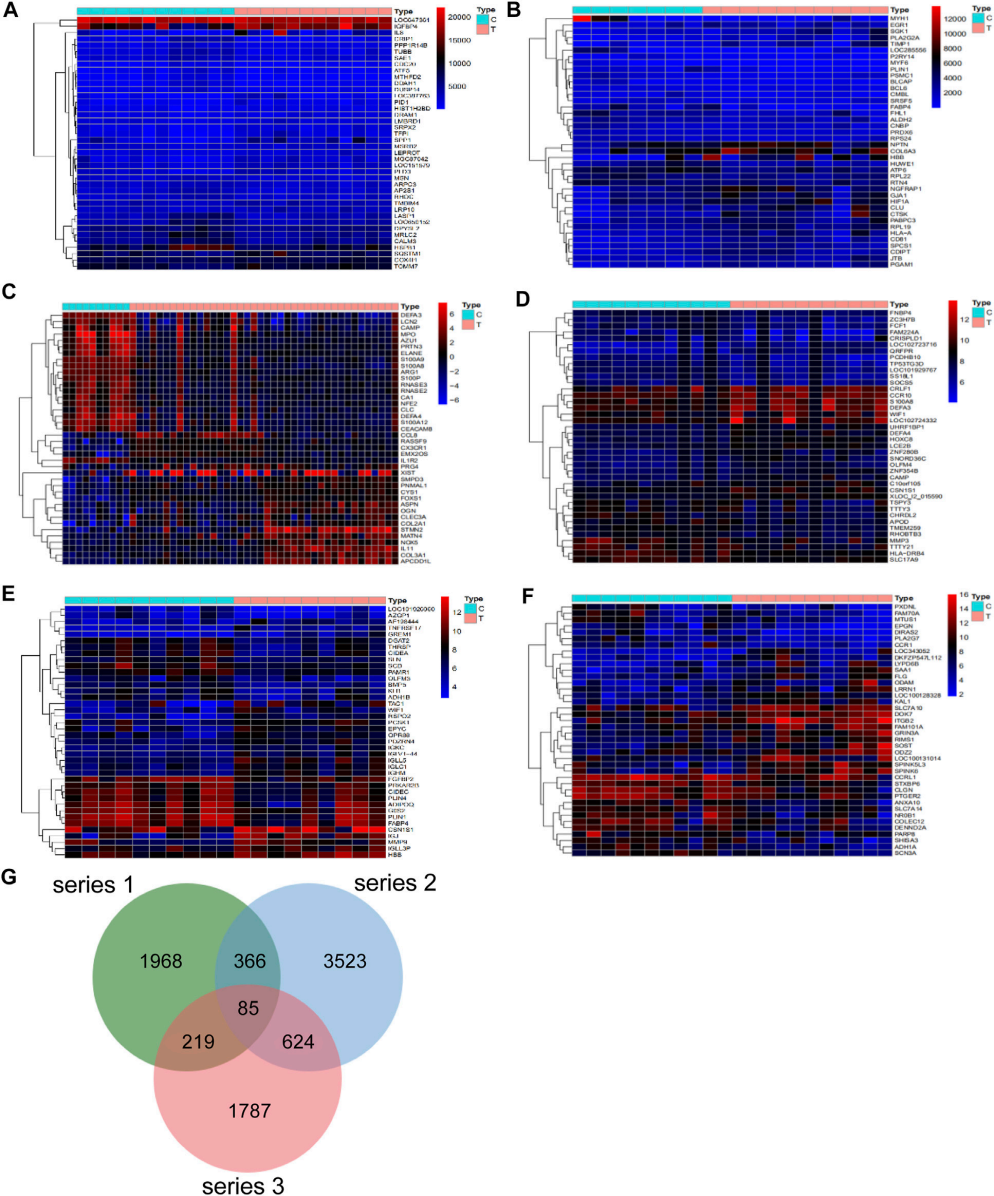
Differentially expressed genes in series 1, series 2 and series 3. The heatmap of series 1 (GSE46750 and GSE82107) **(A,B)**, series 2 (GSE51588 and GSE117999) **(C,D)**, series 3 (GSE32317 and GSE29746) **(E,F)**. **(G)** There are a total of 85 gene intersection in the three series, including PITPNC1, GYPC, AK5, DOK7, KLK3, DRG1, SIGLEC10, PHLDB1, ZCCHC5, TFPI, NUF2, LTC4S, CENPM, RBX1, DKC1, PRPS1, TMEM217, EXO1, ARHGAP11A, ELL2, FANCI, NAMPT, LPPR4, CDK2AP2, RARG, CERCAM, SPTAN1RAB20, MFNG, FKBP10, MARC1, TMEM35, MXD1, ISM1, BGN, SPATA18, FMNL3, LTBP3, CD300C, SPATA19, ABLIM3, RSPO2, STXBP2, CPLX1, ZBTB7B, BCL2L1, PNPLA2, SLC22A4, ANO3, SHROOM2, CRIP2, HPRT1, HBB, PRUNE2, SCRG1, WISP2, CCDC86, PDZRN4, HTR2A, RNF150, PCSK1, ZFP14, CCNB1, ANGPTL2, ZFAND5OGN, SLC11A1, KCNJ15, P2RY14, FABP4, SLC2A1, ALOX12, SF3A3, TCF7, TUSC3, C15orf26, SPATA17, LCAT, SLC16A6, SMOC2, LIMK2, CCDC136, PLA2G5, CKS2 and CCR5.

Differential analysis of the GEO database gene series GSE46750, GSE82107, GSE51588, GSE117999, GSE32317 and GSE29746 identified 538, 2,161, 4,531, 93, 433, and 2341 OA-related targets respectively. The heatmaps and volcano plot of OA-related targets are depicted in Figure 1A–1F and Supplementary Figure S1A–1F. A total of 2,638, 4,598 and 2715 OA-related targets were identified from series 1, 2 and 3, respectively. Altogether there were 85 OA-related targets at the intersection of series 1, 2 and 3 (Figure 1G).

**FIGURE 2:**
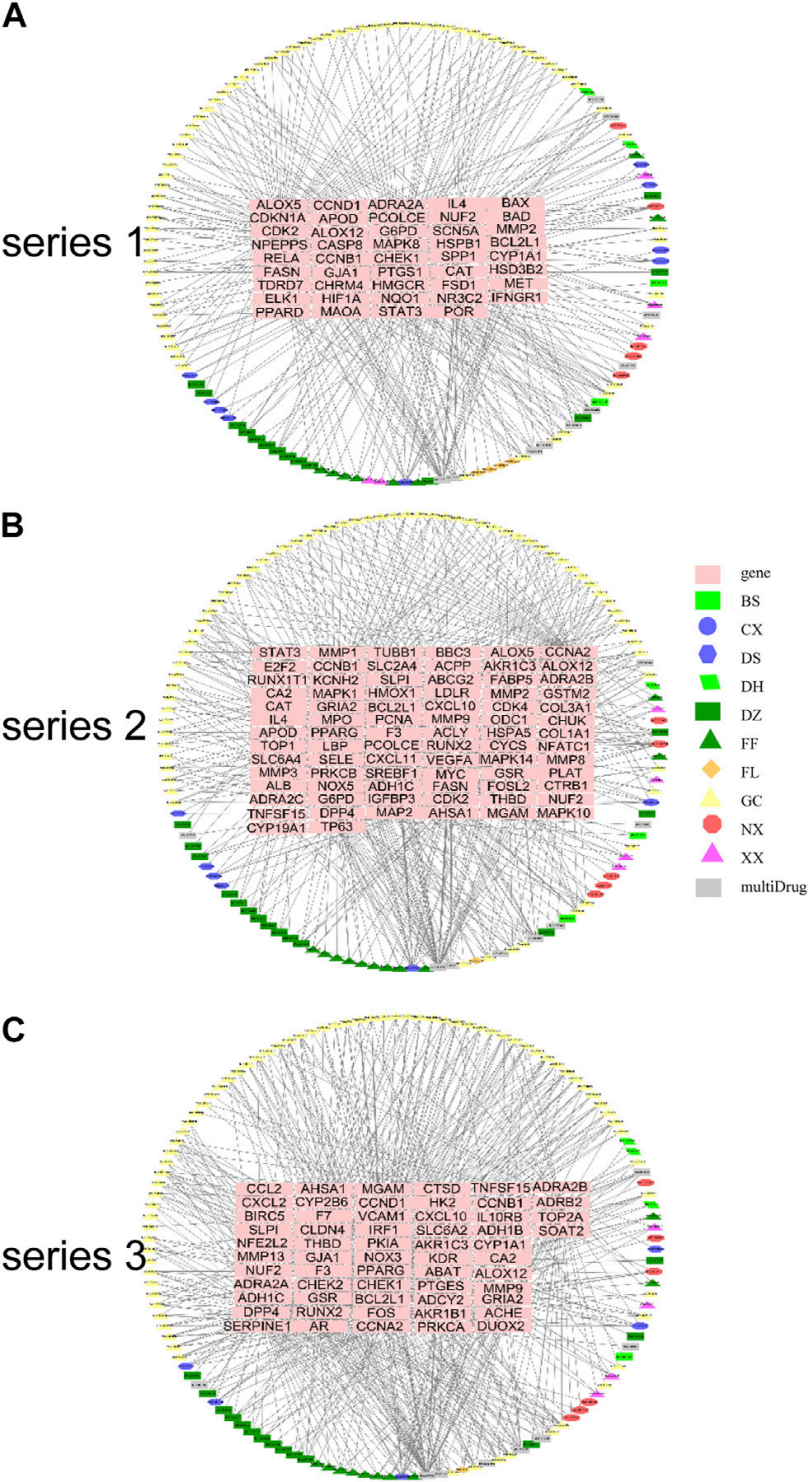
Compound-target network for DHJSD. The pink rectangle represents targets; the green rectangle, blue ellipse, blue hexagon, green parallelogram, cyan rectangle, cyan triangle, orange diamond, yellow triangle, red octagon, purple triangle and gray rectangle represent the compounds from BS, CX, DS, DH, DZ, FF, FL, GC, NX, XX and 22 multi-Drug.

Using the screened compounds and their targets, the compound-target network for DHJSD was constructed (Figure 2). For series 1, the network consisted of 175 nodes (131 compounds for DHJSD and 44 compound targets) and 358 edges (Figure 2A). For series 2, the network consisted of 201 nodes (121 compounds for DHJSD and 80 compound targets) and 486 edges (Figure 2B). For series 3, the network consisted of 188 nodes (129 compounds for DHJSD and 59 compound targets) and 563 edges (Figure 2C).

#### Screening of Candidate Targets for DHJSD Against OA

**FIGURE 3:**
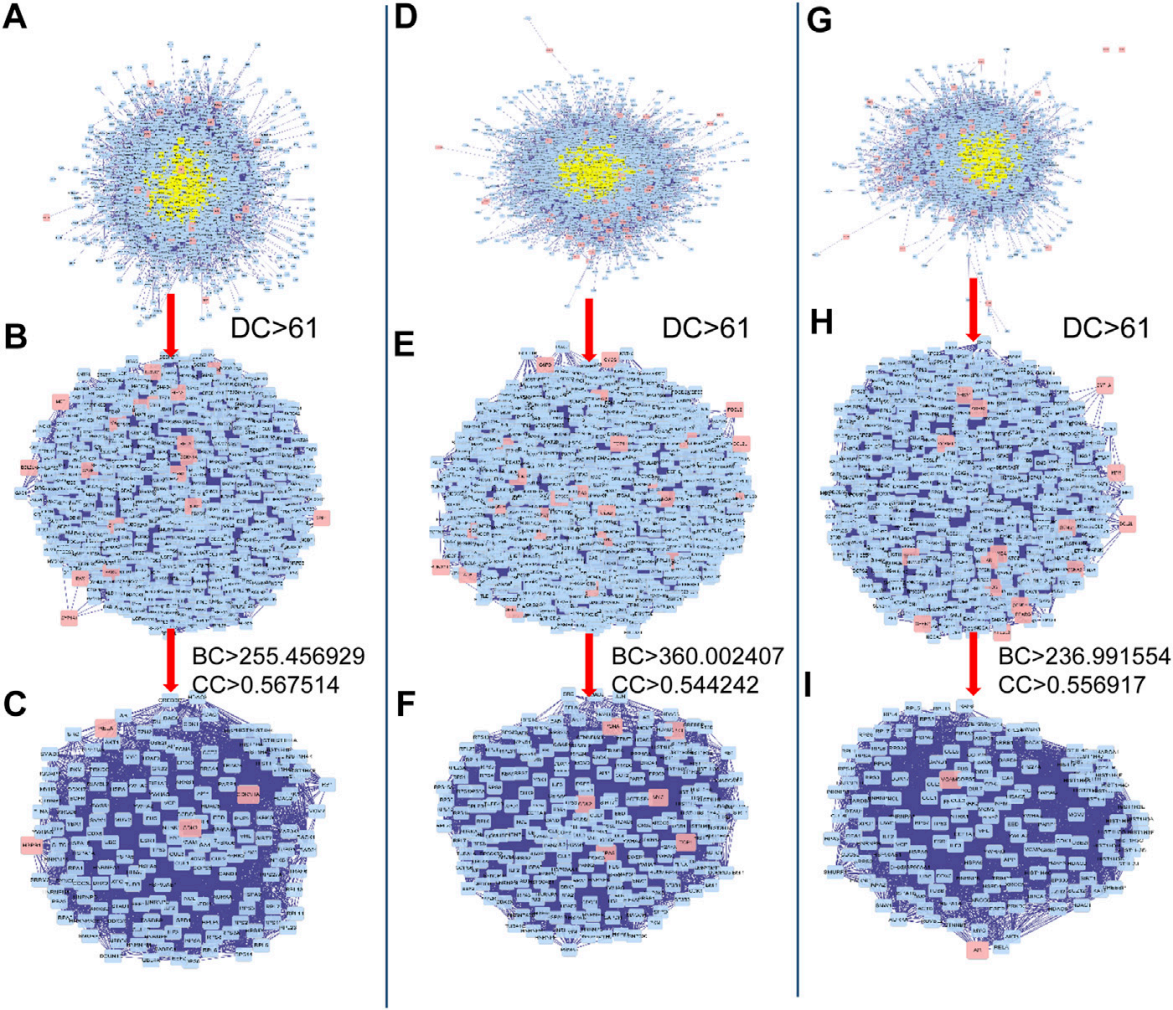
The candidate targets for DHJSD activity against OA. The interactive PPI network topology analysis of DHJSD putative targets and OA-related targets were constructed for series 1 **(A, B, C)**, series 2 **(D, E, F)** and series 3 **(G, H, I)**. DC, degree centrality; BC, betweenness centrality; CC, closeness centrality.

To investigate the possible mechanisms that correlated with the effect of DHJSD on OA, the PPI networks of the OA-related targets and putative DHJSD targets were merged to identify a set of candidate targets. The first network of series 1 consisted of 2,785 nodes and 64,777 edges (Figure 3A), with a median of 27° among all nodes, and identified nodes with more than 61° as significant targets. A secondary network consisting of significant (DC > 61) targets from the first network, which contained 635 nodes and 24,605 edges (Figure 3B). The median values for BC and CC were 255.456929 and 0.567514, respectively. In order to construct the final network, additional screened to identify candidate targets, 163 targets with BC > 255.456929 and CC > 0.567514 were selected (Figure 3C). A total of 163 target genes were eventually identified for DHJSD activity against OA in series 1. The same process was used for series 2, and the network consisted of 3,652 nodes and 90,109 edges (Figure 3D), with a median of 29° among all nodes. The secondary network consisted of 898 nodes and 38,683 edges (Figure 3E). The median values for BC and CC were 360.002407 and 0.544242, respectively. In order to construct the final network, additional screened to identify candidate targets, 228 targets with BC > 360.002407 and CC > 0.544242 were selected (Figure 3F). Thus, 228 target genes were eventually identified for DHJSD activity against OA in series 2. For series 3, the network consisted of 2,462 nodes and 57,944 edges (Figure 3G) with a median 27° among all nodes. The secondary network contained 580 nodes and 23,359 edges (Figure 3H). The median values for BC and CC were 236.991554 and 0.556917, respectively. In order to construct the final network, additional screened to identify candidate targets, 150 targets with BC > 236.991554 and CC > 0.556917 were selected (Figure 3I). Finally, 150 target genes were eventually identified for DHJSD activity against OA in series 3.

#### KEGG and GO Enrichment Analysis of Compound Target Genes

GO and KEGG pathway analysis was conducted to examine the 44 compound targets from series 1, 80 compound targets from series 2 and 59 compound targets from series 3. For series 1, 790 GO terms were significantly enriched (FDR <0.05) (734 BP categories, 14 CC categories and 42 MF categories). The highly enriched GO terms for BP, CC and MF included response to alcohol, response to antibiotic, cyclin-dependent protein kinase holoenzyme complex, mitochondrial outer membrane, NADP binding and histone kinase activity. For series 2, 695 GO terms were significantly enriched (FDR <0.05) (616 BP categories, 32 CC categories and 47 MF categories). The highly enriched GO terms for the BP, CC, and MF included cellular response to oxidative stress, response to oxidative stress, secretory granule lumen, cytoplasmic vesicle lumen, serine-type endopeptidase activity and serine-type peptidase activity. For series 3, 607 GO terms were significantly enriched (FDR <0.05) (551 BP categories, 19 CC categories and 37 MF categories). The highly enriched GO terms for the BP, CC, and MF included regulation of wound healing, regulation of response to wounding, apical part of cell, collagen-containing extracellular matrix, adrenergic receptor activity and histone kinase activity. The top 20 terms are depicted in Supplementary Figures S2–S4.

**FIGURE 4:**
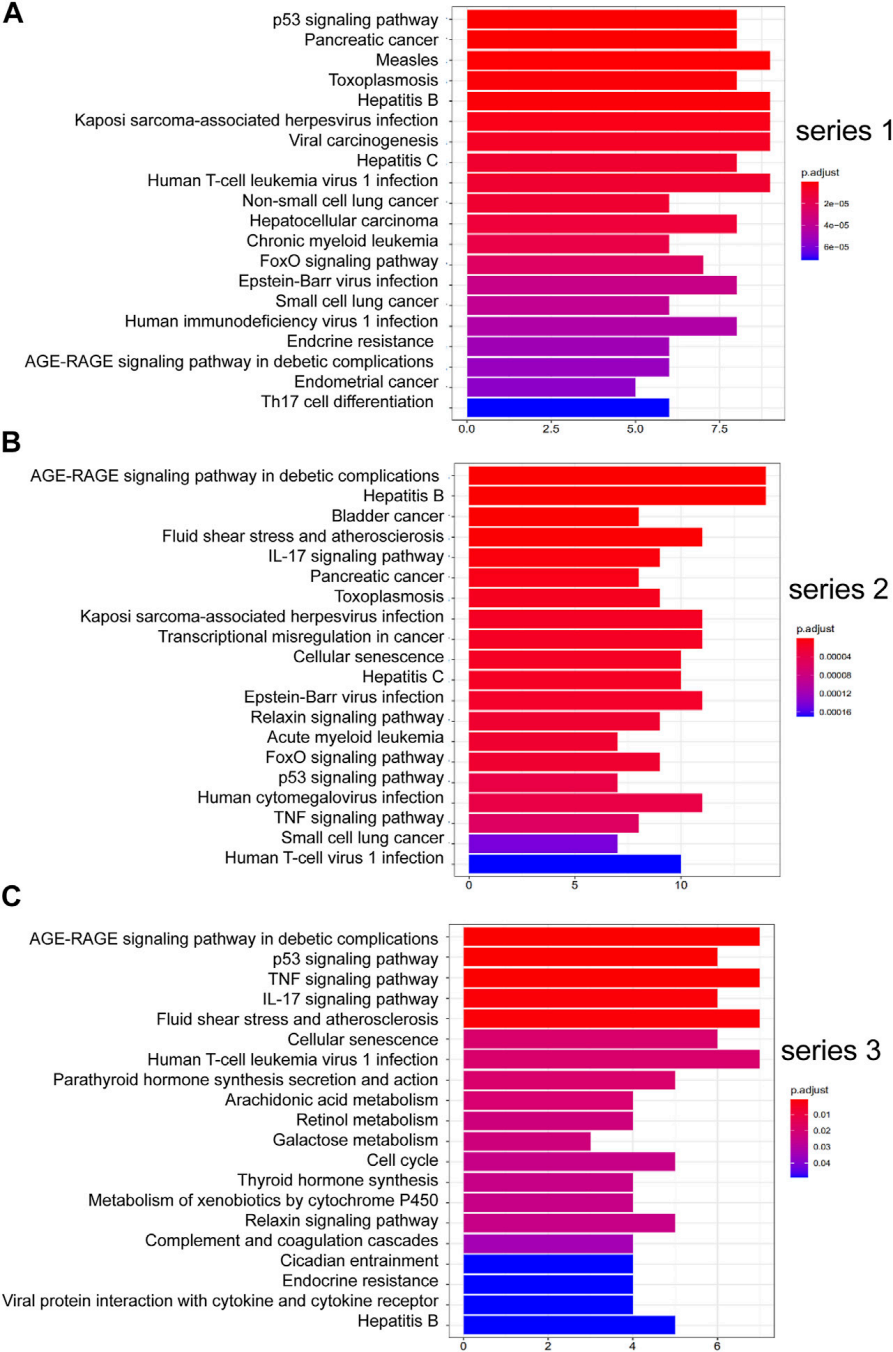
Top 20 KEGG pathway enrichment candidate targets for DHJSD activity against OA for each expression profile. Pathways with significant changes (FDR <0.05) were identified. KEGG pathway labels the vertical axis, and number of differentially expressed genes marks the horizontal. The colour of the bar graph indicates the significance of the enriched KEGG pathway, and the colour gradient represents the size of the *p*-value. Inflammatory signalling pathways are underlined with a red bar. **(A)** series 1, **(B)** series 2 and **(C)** series 3.

Figure 4 shows the pathways significantly correlation with DHJSD and OA, which were identified by the KEGG pathway analysis. For series 1, 93 considerably enriched pathways (FDR <0.05) were identified, which include the p53 signalling pathway, Pancreatic cancer, PI3K-Akt signalling pathway, IL-17 signalling pathway, Toll-like receptor signalling pathway, MAPK signalling pathway and TNF signalling pathway. For series 2, 88 significantly enriched pathways (FDR <0.05) were identified, which include the AGE-RAGE signalling pathway in diabetic complications, IL-17 signalling pathway, p53 signalling pathway, TNF signalling pathway, Toll-like receptor signalling pathway, PI3K-Akt signalling pathway, MAPK signalling pathway, NF-κB signalling pathway and Ras signalling pathway. For series 3, 21 significantly enriched pathways (FDR <0.05) were identified, which include the AGE-RAGE signalling pathway in diabetic complications, p53 signalling pathway, TNF signalling pathway and IL-17 signalling pathway. Series 1 and 2 contained 72 identical pathways, series 2 and 3 contained 14 identical pathways and series 1 and 3 consisted of 13 identical pathways.

#### Gene Pathway Network Analysis

**FIGURE 5:**
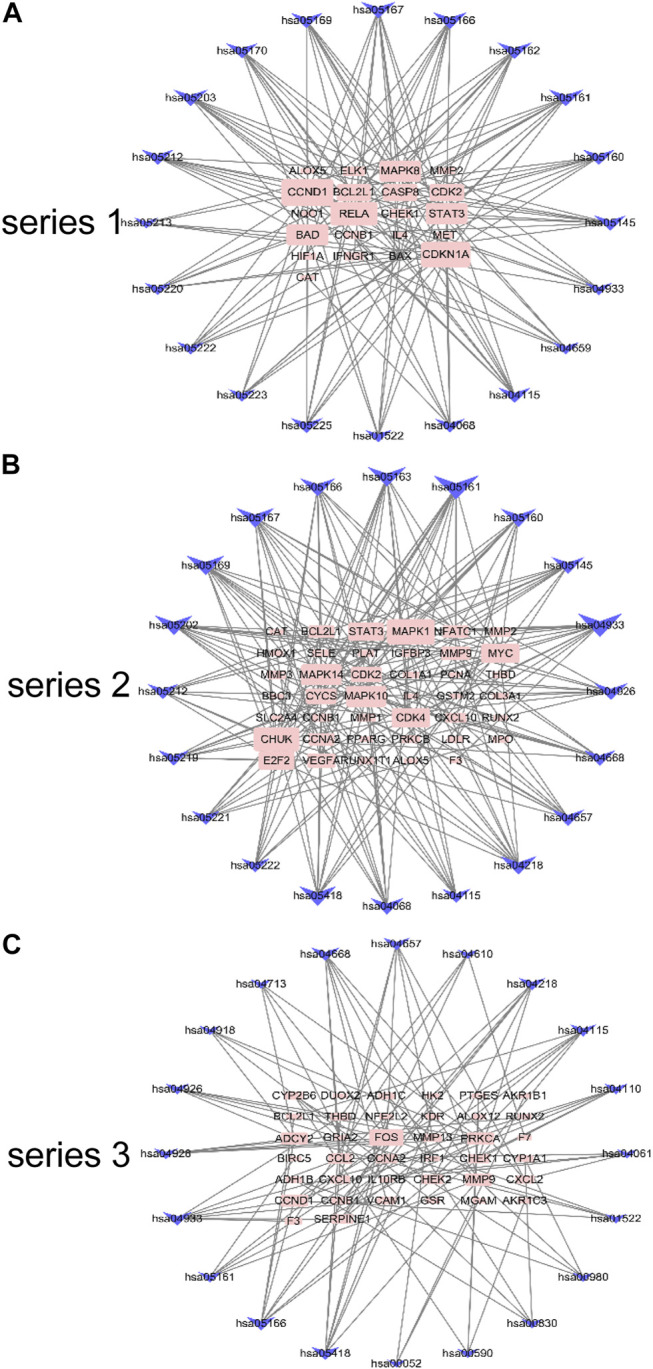
Gene pathway network for DHJSD activity against OA. Top 20 pathways was performed with degree according to the topological analysis for series 1**(A)**, series 2**(B)** and series 3**(C)**.

The gene pathway network based on the significantly enriched pathways and genes were constructed (Figure 5). For series 1, based on the topological analysis and degree 20 pathways was performed. The network diagram showed that MAPK8, CCND1, RELA and CDKN1A had the highest degree, and selected as the core target gene. Moreover, BAD, STAT3, BCL2L1, CASP8 and CDK2 also exhibited a large degree (Figure 5A). For series 2, based on the topological analysis and degree 20 pathways was performed. The network diagram showed that MAPK1, MAPK14, MAPK10, CHUK had the highest degree, and selected as the core target gene. Moreover, STAT3, MMP9, MYC, CDK2, CYCS, CDK4 and E2F2 also exhibited a large degree (Figure 5B). For series 3, based on the topological analysis and degree 20 pathways was performed. The network diagram indicated that FOS had the highest degree, and selected as the core target gene. Moreover, ADCY2, MMP13, PRCKA, CCL2, MMP9, CCND1 and SERPINE1 also exhibited a high degree (Figure 5C).

**FIGURE 6:**
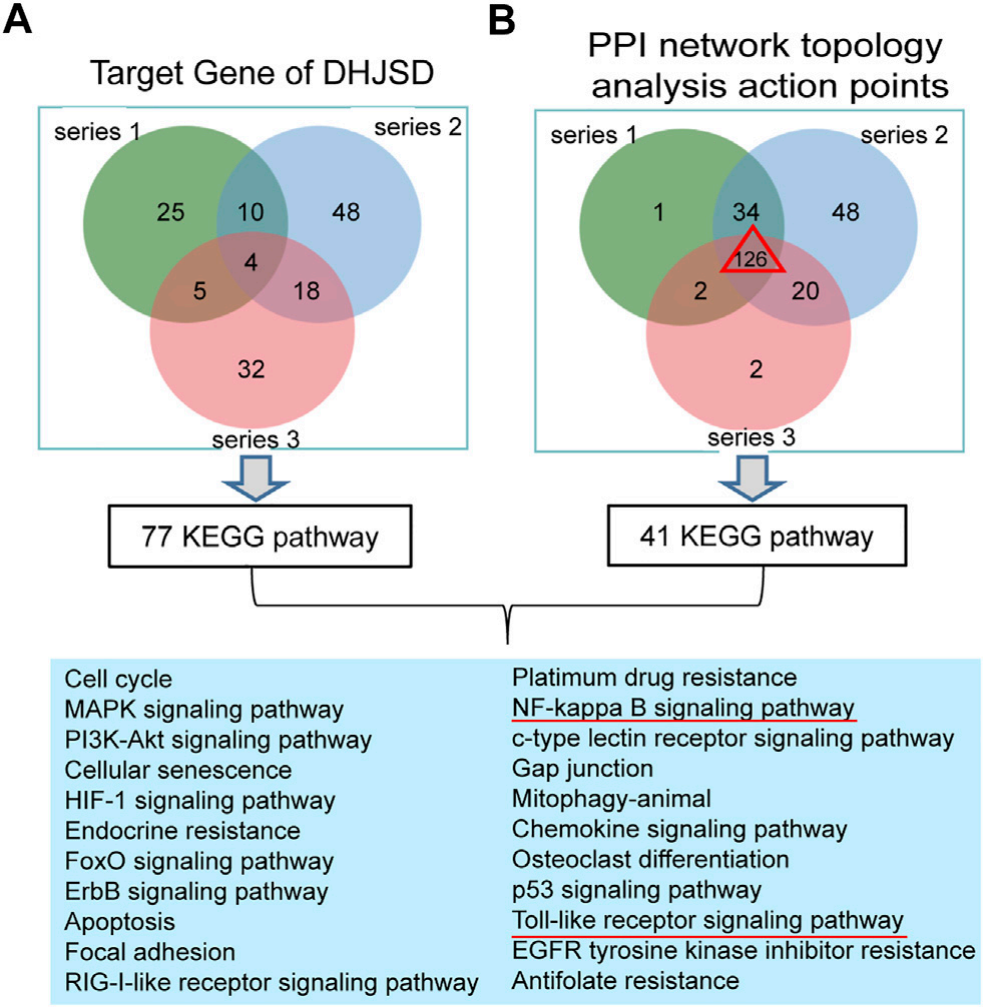
Results of the KEGG enrichment analysis. **(A)** KEGG analysis of the union of the three series target genes of DHJSD, 77 KEGG pathways were significantly enriched. **(B)** KEGG analysis of the Venn of three series’ PPI network topology analysis action points. The result showed that there are a total of 126 genes and 41 KEGG pathways. Venn analysis showed that 22 KEGG pathways were identified after the intersection of A and B.

The intersection of DHJSD and the three chip targets and the core targets obtained after the topological analysis of the three chips were selected for KEGG analysis. DHJSD was analysed for the union of the three chip targets and 77 KEGG pathways were detected. After the topological analysis of the three chips, 126 core targets were selected for KEGG analysis to obtain 41 KEGG pathways. The intersection of 77 and 41 pathways and 22 KEGG pathways are shown in Figure 6. Based on these results, the NF-κB signalling pathway and the toll-like receptor signalling pathway were chosen as the key pathways for further research.

#### Molecular Docking Analysis

**FIGURE 7:**
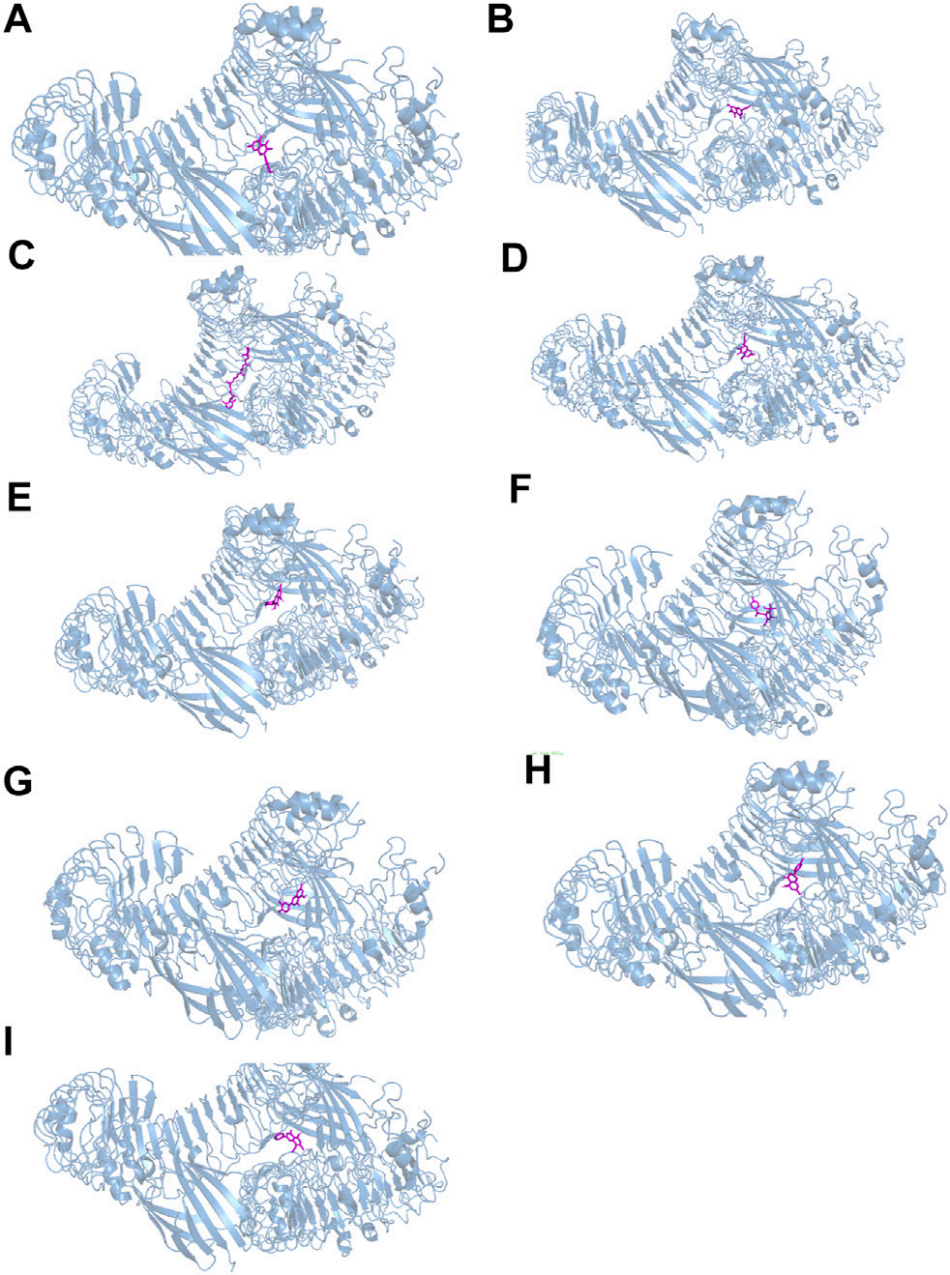
The docking between the top nine compounds [quercetin **(A)**, baicalein **(B)**, beta-carotene **(C)**, isorhamnetin **(D)**, kaempferol **(E)**, licochalcone a **(F)**, luteolin **(G)**, naringenin **(H)** and wogonin **(I)**]of Duhuo Jisheng Decoction (DHJSD) and Osteoarthritis target TLR4. The crystal structures of TLR4 was shown in blue and the compound was shown in purple.

The results of molecular docking analysis to confirm the top nine compounds (quercetin, baicalein, beta-carotene, isorhamnetin, kaempferol, licochalcone a, luteolin, naringenin and wogonin) related to TLR4 showed that all compounds had strong binding affinity to TLR4, as shown in Table 2. The top three compounds strongly associated with TLR4 were beta-carotene, quercetin and luteolin (Figure 7). In addition, the top nine compounds also showed tightly bound to RELA (also known as NF-κB3), CHUK (also known as IKKα) (Supplementary Table S2, Figure S5, S6), RELA and CHUK were the core target genes obtained from the topological analysis. Verification of the involvement of targeted pathways in the therapeutic effects of DHJSD against OA.

**TABLE 2:** The binding free energy between the top nine compounds and TLR4.

Compound	Molecular formula	CAS	docking score (kcal/mol)
Quercetin	C_15_H_10_O_7_	117-39-5	−8.1
Baicalein	C_15_H_10_O_5_	491-67-8	−7.5
Beta-carotene	C_40_H_56_	7,235-40-7	−8.3
Isorhamnetin	C_16_H_12_O_7_	480-19-3	−7.7
Kaempferol	C_15_H_10_O_6_	520-18-3	−7.6
Licochalcone a	C_21_H_22_O_4_	58749-22-7	−7.0
Luteolin	C_15_H_10_O_6_	491-70-3	−8.0
Naringenin	C_15_H_12_O_5_	480-41-1	−7.6
Wogonin	C_16_H_12_O_5_	632-85-9	−7.4

#### DHJSD Inhibited the Expression of miR-146a-5p and miR-34a-5p in LPS-Exposed Chondrocytes

**FIGURE 8:**
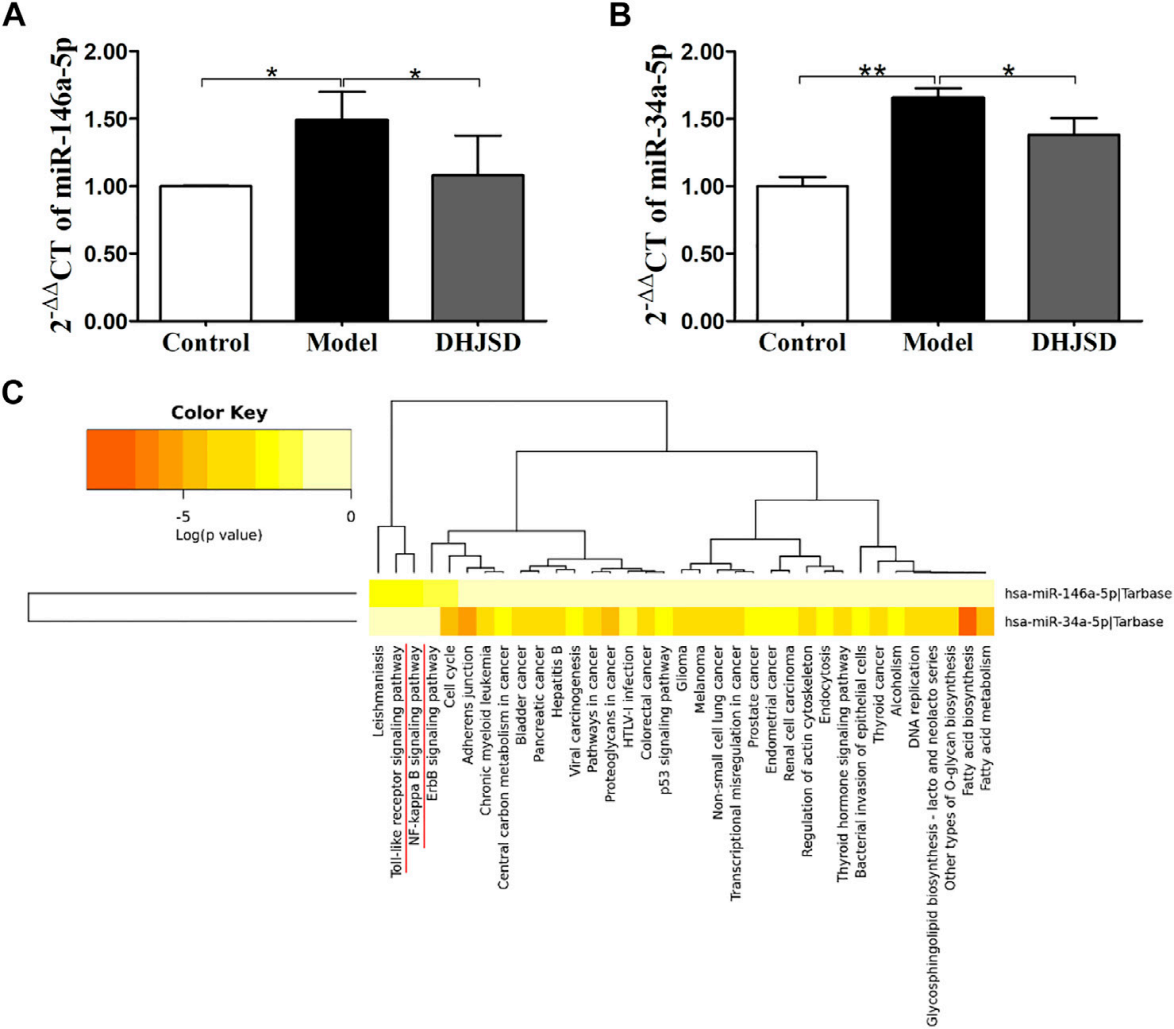
DHJSD suppresses the mRNA expression of miR-146a-5p **(A)** and miR-34a-5p **(B)**. Chondrocytes were treated with or without LPS and DHJSD for 8 h. The expressions of miR-146a-5p and miR-34a-5p were upregulated after LPS exposure and downregulated after DHJSD treatment. *β*-actin was selected as internal standards. miRNA-regulated gene-pathway analysis **(C)** found that the toll-like receptor signalling pathway and the NF-κB signalling pathway were significantly enriched. Values are mean ± standard deviation (SD), and SD is shown as vertical bars. ^**^ indicates *p* < 0.01 and ^*^ indicates *p* < 0.05 compared with control or LPS-exposed chondrocytes.

LPS exposure increased the expressions of miR-146a-5p (Figure 8A) and miR-34a-5p (Figure 8B), whereas DHJSD downregulated these expressions in LPS-exposed chondrocytes. The miRNA-regulated gene-pathway analysis (Figure 8C) showed that the toll-like receptor signalling pathway (*p* = 0.012) and the NF-κB signalling pathway (*p* = 0.042) were significantly enriched, which was consistent with the NPA analysis.

#### DHJSD Suppressed the Expression of Inflammatory Proteins and Genes

**FIGURE 9:**
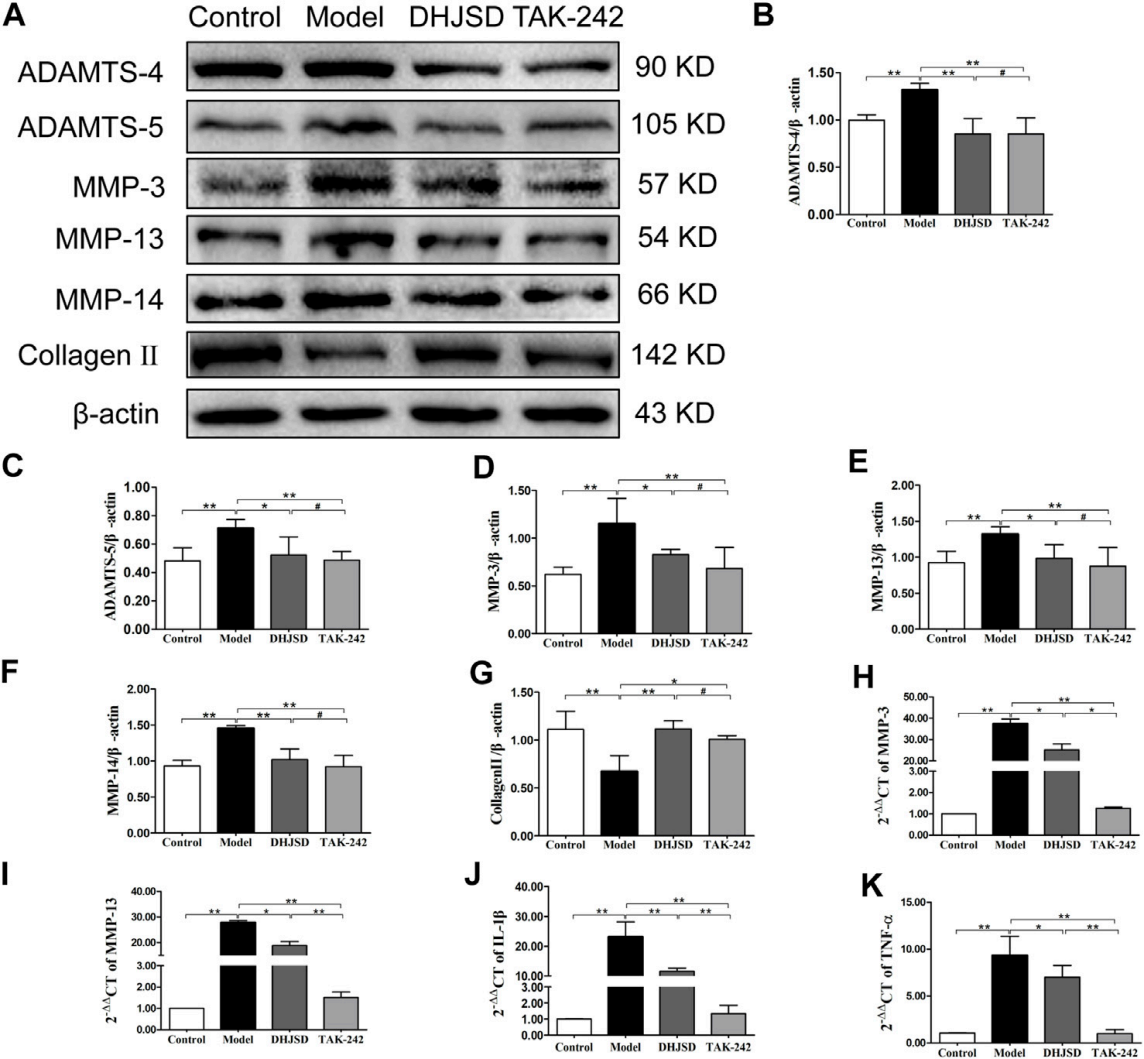
DHJSD suppresses the expression of inflammatory proteins and genes. Chondrocytes were cultivation with or without LPS and DHJSD or TAK-242 treatment in the presence of LPS for 8 h, and the associated protein level was conformed by western blot analysis **(A)**. Protein levels of ADAMTS-4 **(B)**, ADAMTS-5 **(C)**, MMP-3 **(D)**, MMP-13 **(E)**, MMP-14 **(F)** and collagen-II **(G)**. β-actin was used as the internal control for normalisation. MMP-3 **(H)**, MMP-13 **(I)**, IL-1β **(J)** and TNF-α **(K)** mRNA expressions were detected by RT-PCR. GAPDH was selected as internal standards. Values are mean ± standard deviation (SD), and SD is shown as vertical bars. *N* = 3. ^★★^Indicates *p* < 0.01 and ^★^indicates *p* < 0.05 compared with control or LPS-exposed chondrocytes. ^#^Implies *p* > 0.05 compared with TAK-242.

The expression of collagen Ⅱ protein was upregulated by DHJSD or TAK-242. Chondrocytes were treated with or without LPS and DHJSD and TAK-242 in the presence of LPS for 8 h (Figure 9A). The protein expressions of ADAMTS-4, ADAMTS-5, MMP-3, MMP-13 and MMP-14 were downregulated by DHJSD or TAK-242 treatment (Figures 9B–F). The mRNA expressions of MMP-3, MMP-13, IL-1β and TNF-α upregulated after LPS treatment and downregulated after DHJSD or TAK-242 treatment (Figures 9I–K).

#### DHJSD Effects Were Associated With the TLR4/MyD88/NF-κB Pathway

**FIGURE 10:**
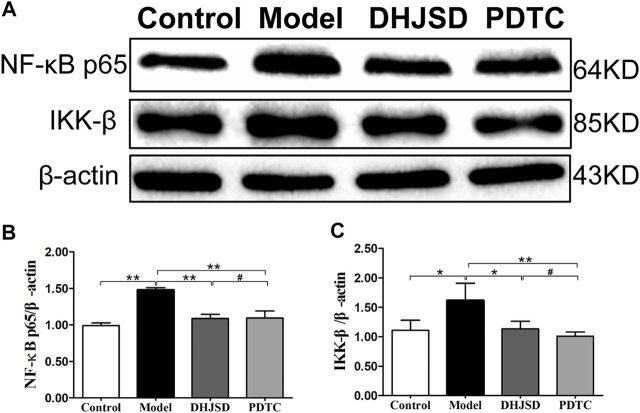
DHJSD administration effects the NF-κB signalling pathway genes in LPS-exposed chondrocytes. The protein expression of NF-κB p65 and IKK-β after treatment with different concentrations of DHJSD for 8 h. Western blot analysis of protein expression levels **(A)**; the protein expressions of NF-κB p65 **(B)** and IKK-β **(C)** were significantly upregulated after LPS exposure and downregulated after DHJSD or PDTC treatment. Data are expressed as mean ± standard deviation (SD), and SD is shown as vertical bars. N = 3. ^★^Indicates *p* < 0.05, ^★★^Indicates *p* < 0.01, ^#^implies *p* > 0.05 compared with PDTC.

**FIGURE 11:**
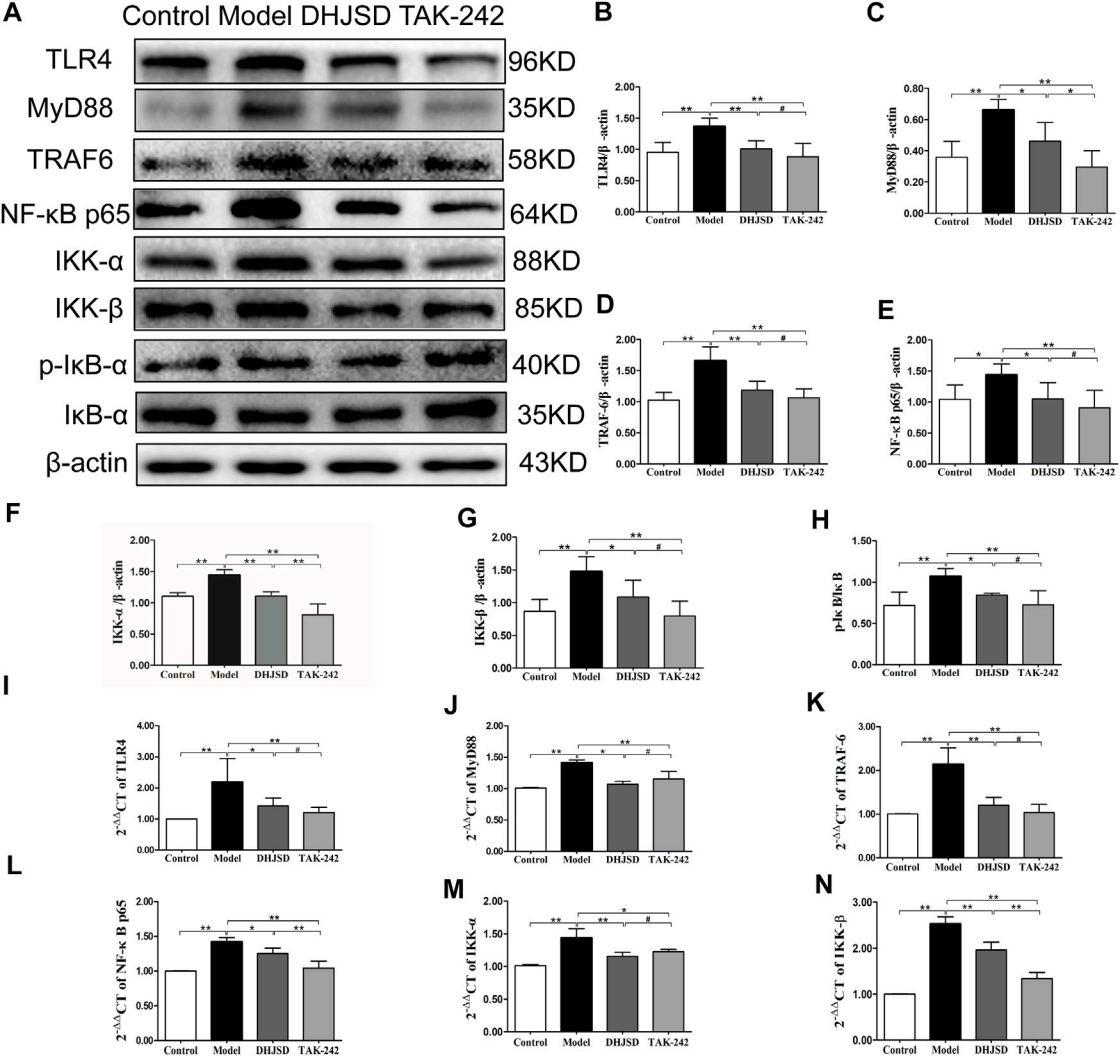
DHJSD inhibits the TLR4 signalling pathway-induced apoptosis of LPS-exposed chondrocytes. Chondrocytes were cultivation with or without LPS and DHJSD or TAK -242 treatment in the presence of LPS for 8 h, and the associated protein level was tested by western blot **(A)**. Protein levels of TLR4 **(B)**, MyD88 **(C)**, TRAF-6 **(D)**, NF-κB p65 **(E)**, IKK-α **(F)**, KK-β **(G)** and *p*-IκB-α **(H)**. *β*-actin was selected as internal standards. TLR4 **(I)**, MyD88 **(J)**, TRAF-6 **(K)**, NF-κB p65 **(L)**, IKK-α **(M)** and IKK-β **(N)** mRNA expressions were detected by qPCR. GAPDH was selected as internal standards. Values are means ± standard deviation (SD), and SD is shown as vertical bars. ^**^indicates *p* < 0.01 and ^*^indicates *p* < 0.05 compared with control or LPS-exposed chondrocytes. ^#^ implies *p* > 0.05 compared with TAK-242.

All the biomarkers of the LPS-exposed cells exhibited significant differences compared with intact cells. The protein expressions of NF-κB p65 and IKK-β were significantly upregulated after LPS exposure but significantly downregulated under DHJSD and PDTC treatment (Figures 10A–C). In the DHJSD and TAK-242 treatment groups, the protein expressions of TLR4, MyD88, TRAF-6, NF-κB p65, IKK-α, IKK-β and *p*-IκB-α were downregulated, and the mRNA expressions of TLR4, MyD88, TRAF-6, NF-κB p65, IKK-α and IKK-βwere downregulated (Figure11). In the DHJSD or PDTC treatment groups, the protein expressions of NF-κB p65 and IKK-β were downregulated, and the mRNA expressions of NF-κB p65 and IKK-β were downregulated (Figure 10). These changes have close correlation with changes in the TLR4/MyD88/NF-κB pathway mediated chondrocyte apoptosis, indicating that chondrocyte inflammation is a vital mechanisms of the effects of DHJSD.

## Discussion

In this research, we confirmed the utility of NPA for investigating the molecular targets of a complex herbal formulation using two sequential experiments. DHJSD is a typical widely-used complex herbal formulation with complicated ingredients (15 herbs), and research involving DHJSD is limited. We identified the quality control of DHJSD using conventional HPLC and identified 206 candidate compounds related to OA by TCM. In experiment 1, we constructed a standard NPA for DHJSD. The KEGG and GO enrichment analyses identified several inflammatory signalling pathways highly associated with DHJSD, and the results of Venn analysis between the target gene and PPI network topology analysis action point supported that the NF-κB signalling pathway and the toll-like signalling pathway play a key role in the effects of DHJSD, which were then subjected to experiment 2. We confirmed the involvement of miR-146a-5p and miR-34a-5p, the TLR4/MyD88/NF-κB signalling pathway and inflammatory-related genes by western blotting and qPCR. We found that DHJSD in fact contributed suppression of the expression of miR-146a-5p and miR-34a-5p, the TLR4/MyD88/NF-κB signalling pathway-related genes and inflammatory-related genes, which were upregulated upon LPS exposure. Hence, the role of the TLR4/MyD88/NF-κB signalling pathway was confirmed. In the present study we first performed conventional experiments combined with NPA and obtained an intact chain of evidence of DHJSD; thus, the value of NPA emerged.

### Quality Control of DHJSD

Ligustrazine hydrochloride, paeoniflorin and osthole were chosen to be the quality control markers for DHJSD extract since it have been confirmed as quality control markers of CX, BS and DH, respectively, according to the pharmacopoeia of the People’s Republic of China (2015 version). The results of HPLC identified ligustrazine hydrochloride*,* paeoniflorin and osthole, confirming the three compounds as quality control markers for DHJSD extract. We identified 206 candidate compounds from the NPA analysis that may be associated with DHJSD activity against OA. Only paeoniflorin was one of these candidate compounds which may play a major role in the treatment of DHJSD against OA. The OB values of paeoniflorin was 53.87 and DL values was 0.79. Whereas the candidate compounds which excludes ligustrazine hydrochlorideand and osthole. The experimental result indicated that the pharmacological quality control markers from the pharmacopoeia may be a stable components of DHJSD but have some limitations, which were not present in the list of effective compound from NPA. Hence, in our next experiment the core bioactive compounds from NPA may considered as new candidate markers.

### Bioactive Components and Molecular of DHJSD Construction by NPA Analysis

In this study, we first used three OA-related gene series for NPA. The compound-target networks of DHJSD were constructed using 131, 121 and 129 compounds and 44, 80 and 59 compound targets in the three series, respectively. Quercetin, luteolin, wogonin and baicalein acted on 23, 11, 9 and 8 targets in series 1; quercetin, luteolin, baicalein, and kaempferol acted on 41, 18, 13 and 12 targets in series 2; and quercetin, luteolin, kaempferol and wogonin acted on 36, 14, 12 and 10 targets in series 3, respectively. Therefore, quercetin, luteolin and kaempferol were probably the vital pleiotropically active compounds for DHJSD.

The candidate targets for DHJSD activity against OA were obtained by merging the three series PPI networks of DHJSD putative targets and OA-related targets. Furthermore, to achieve more accurate targets and structure a new network, we set 3, 6, 5 parameters, including DC and BC to further screen the nodes. Finally, 163, 228 and 150 targets were identified in series 1, 2 and 3, respectively, with 85 common action point targets.

To investigate the biological information of the targets of DHJSD activity against OA, we performed GO analysis. Results showed that DHJSD was involved in the regulation of some biological processes, indicating that the reduction of cartilage degeneration by DHJSD might related to the inflammatory response. The three series target genes related to the gene pathway network was constructed, and Venn analysis was performed to identify the intersections of the three OA-related pathway to identify key pathways for DHJSD activity against OA. The results of the gene pathway network analysis suggested that MAPK8, MAPK1 and FOS had the maximum degree, and it might be the core target in series 1, 2 and 3. The other top three genes were CCND1, RELA and CDKN1A in series 1; MAPK14, MAPK10 and CHUK in series 2; and ADCY2, MMP13 and PRCKA in series 3. In our previous study, the level of MMP-9 in LPS exposed chondrocytes was upregulated, and DHJSD reduced the MMP-9 level. MMP-9 was the key target genes in series 2 and series 3; furthermore the MMP family of genes, including MMP-1, MMP-2, MMP-3 and MMP-13, were also the key target genes of DHJSD in all the series. The MMP family participates in degradation of the extracellular matrix and basement membrane such as collagen Ⅱ and collagen Ⅳ, which leads to joint integrity and function (Lewis et al., 1997; Van Doren, 2015). Further verification is needed in future investigation.

### Verification of the Key Role of the NF-κB Signalling Pathway of DHJSD in OA

Inflammation has an important and close relationship with the pathogenesis of OA (Shen et al., 2017). MMP-9 is one of the cartilage degradation biomarkers that is directly affected. Previous research has shown that the expression of MMP-9 is under the control of NF-κB signalling pathway (Alves et al., 2014; Nambi, 2021). In our experiments, the MMP-9 levels were significantly increased after LPS exposure, consistent with our previous study (Xu et al., 2021), but remarkably decreased after DHJSD treatment (Xu et al., 2019). NF-κB p65 and IKK-β expressions were significantly upregulated after LPS exposure and downregulated after DHJSD or PDTC treatment. IKK-β is a vital upstream activator that participates in the active and release cytosolic sequestration of NF-κB p65 subunits and nuclear repositioning (Durand and Baldwin, 2017; Jimi et al., 2019). The expression of these genes is altered after DHJSD or PDTC treatment, we hypothesised that the NF-κB signalling pathway plays a distinctive role in the effects of DHJSD on OA.

### Verification of the Key Role of the TLR4/MyD88/NF-κB Signalling Pathway of DHJSD in OA

Regarding the involvement of miR-146a-5p and miR-34a-5p in OA development, Shao et al. found that miR-146a-5p would activated NF-κB signalling pathway through TRAF6 which contribute to the chondrocyte apoptosis (Shao et al., 2020). MiR-34a-5p induced joint destruction and miR-34a-5p ASO injection protection cartilage damage (Endisha et al., 2021). Another recent study also showed that miRNA-regulated neuronal inflammation and apoptosis through NF-κB 1-induced LINC00665, and the inhibition of miR-34a-5p would restrain NF-κB-1 expression (Deng et al., 2021). In addition, the prediction target of miR-146a-5p showed that the NF-κB signalling pathway and the toll-like receptor signalling pathway were closely related pathways. Our experiment showed that after the LPS exposure of chondrocytes, the expressions of miR-146a-5p and miR-34a-5p increased in the OA cell model group. Moreover, the miRNA-regulated gene-pathway analysis revealed that the toll-like receptor signalling pathway and the NF-κB signalling pathway were significantly enriched, which was consistent with the NPA analysis.

The venn analysis between the KEGG pathway enrichment analysis of target gene and PPI network topology analysis action point found 22 identical pathways, and the NF-κB signalling pathway and the toll-like receptor signalling pathway were in the lists of both analysis. The toll-like receptor (TLR) family has primary sensors that are essential for eliciting innate immune responses (Akira et al., 2006). The TLR signalling pathway controls the expression of inflammatory cytokine genes by the activation of NF-κB (Hayden et al., 2006). TLR4 recruits the MyD88 adaptor triggering the MyD88-dependent pathway, and the TLR4 MyD88 complex interacts with TRAF6. Next, IκB kinase is regulated by two kinases IKK-α and IKK-β, followed by phosphorylation and degradation, resulting in NF-κB p65 release from cytosolic sequestration and nuclear repositioning (Wang et al., 2015). Based on the above described results, we finally determined the TLR4/MyD88/NF-κB signalling pathway for further verification in experiment 2, in which we observed the effects of LPS, DHJSD, PDTC and TAK-242 on the expression of proteins related to the TLR4/MyD88/NF-κB signalling pathway. The expressions of NF-κB p65 and IKK-β were downregulated after DHJSD or PDTC treatment. The expressions of TLR4, MyD88, TRAF-6, NF-κB p65, IKK-α, IKK-β, P-IKB-α and IKB-α were affected by DHJSD and TAK-242 treatment, confirming the key role of the TLR4/MyD88/NF-κB signalling pathway in the effects of DHJSD treating OA.

Collagen Ⅱ was significantly downregulated after LPS exposure and increased with DHJSD or TAK-242 treatment, whereas the expressions of ADAMTS-4, ADAMTS-5, MMP-3, MMP-13 and MMP-14 showed opposite results. Collagen II plays a pivotal role in regulating cartilage matrix degeneration in OA progression (Hao et al., 2019), and the synthesis of collagen II would inhibited by MMPs including MMP-3 and MMP-13, while upregulated by IL-1β or other pro-inflammatory cytokines (Kapoor et al., 2011; Fei et al., 2019). MMP-3, MMP-13 and MMP-14 are crucial enzymes that directly degrade the extracellular matrix (Honsawek et al., 2013). ADAMTS-4 and ADAMTS-5 were identified as the major cartilage aggrecanases in arthritis (Cooper et al., 2000). These proteins play vital role in cartilage matrix degeneration, confirming that DHJSD, PDTC or TAK-242 therapy would influence OA development. Furthermore, molecular docking showed that beta-carotene, quercetin and isorhamnetin had strong affinity for TLR4, implying that TLR4 plays an important role in the DHJSD treatment of OA. In addition the top nine compounds also showed strong affinity with TLR4/MyD88/NF-κB-related genes. As proved by the above-result, the TLR4/MyD88/NF-κB-related genes, inflammatory gene transcription and various inflammatory factors function together to regulate the inflammatory reaction in the DHJSD treatment of OA.

Altogether, the results of NPA and conventional experiments suggested that the mechanisms of the action of DHJSD include comprehensive effects of several compounds and pathways, and the TLR4/MyD88/NF-κB signalling pathway plays a key role. Suppression of the inflammatory response involved in the chondrocyte apoptosis, which may relevant to cartilage degeneration, is the key mechanism.

## Conclusion

To explore the molecular targets of DHJSD in treating OA, we used NPA and designed two experiments, including the angles of compounds, genes and pathways. The results of this study suggest that NPA requires a powerful tool to investigate the molecular targets of the complex herbal formulations such as DHJSD and revealed the TLR4/MyD88/NF-κB signalling pathway as a key therapeutic target, although requiring and further investigation.

## Data Availability

The original contributions presented in the study are included in the article/Supplementary Material, further inquiries can be directed to the corresponding author.
