# Supplementary material for: Confirmation of inhibitingTLR4/MyD88/NF-κB Signalling Pathway by Duhuo Jisheng Decoction on Osteoarthritis: A Network Pharmacology Approach-Integrated Experimental Study
Source: Front Pharmacol. 2022 Jan 24;12:784822. doi: 10.3389/fphar.2021.784822 (PMC8818874; doi:10.3389/fphar.2021.784822)
Supplement: Supplementary file 1 [file DataSheet1.docx]

Supplementary Material

**
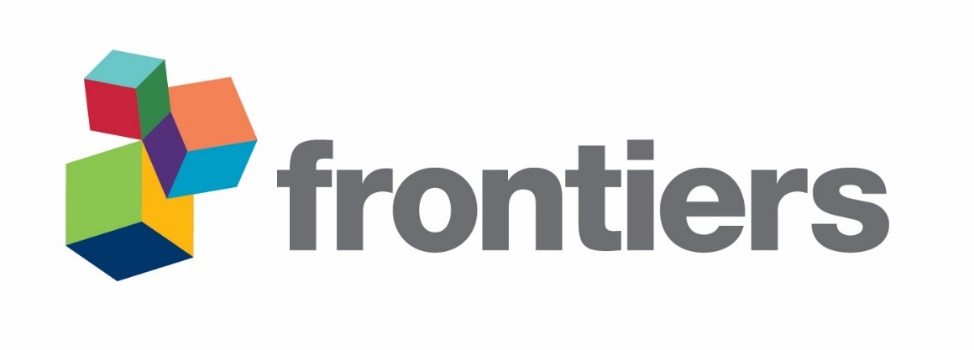
**


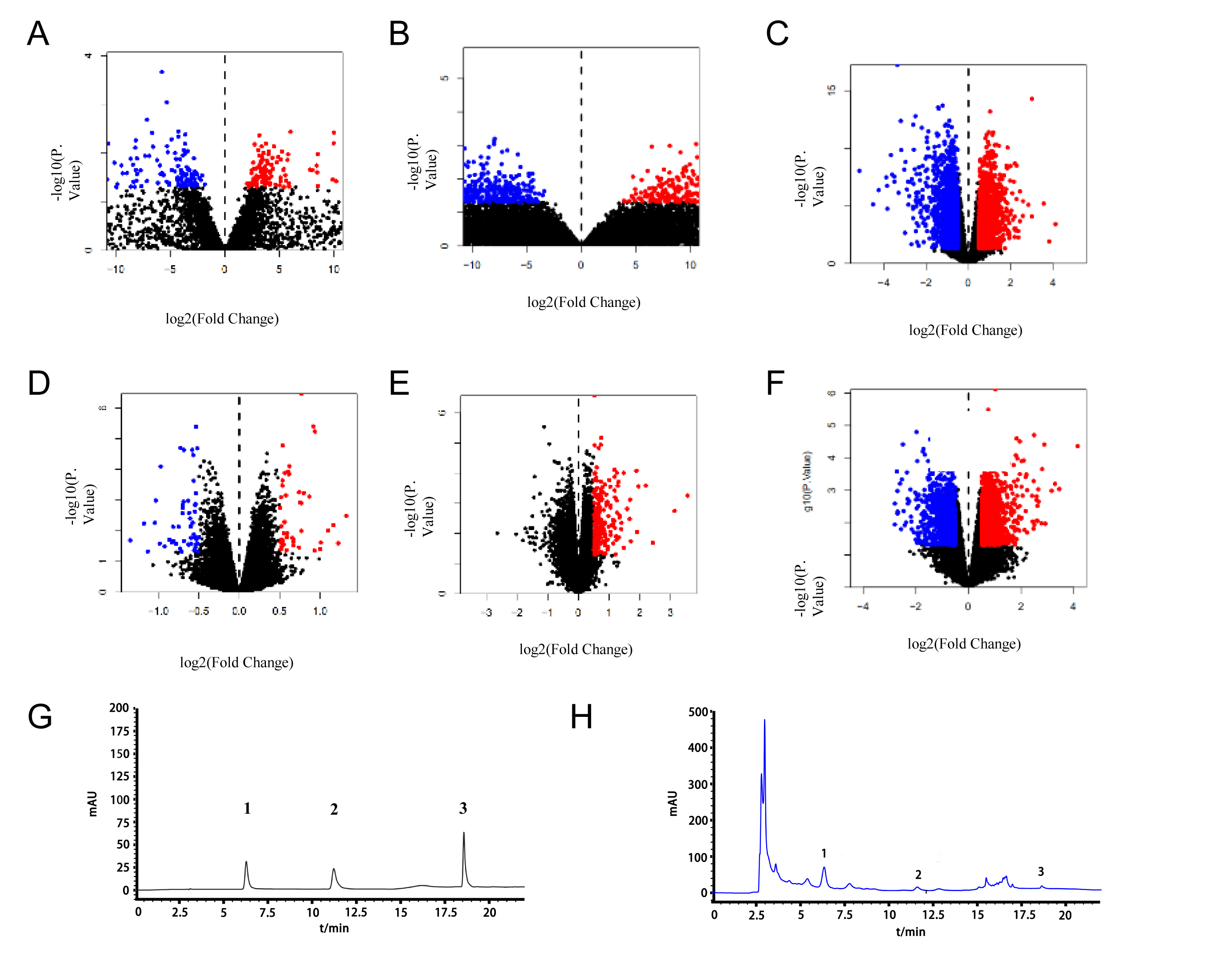


**Supplementary Figure 1.** Differentially expressed genes in series 1, series 2 and series 3. The volcano plot of series 1(GSE46750 and GSE82107) (A, B), series 2(GSE51588 and GSE117999) (C, D) and series 3(GSE32317 and GSE29746) (E, F).

Quality control of DHJSD extracts by high-performance liquid chromatogram (HPLC) assay. G. The liquid chromatogram of the reference substance was composed of three peaks at 230 nm: Peak 1: paeoniflorin*,* Peak 2: ligustrazine hydrochloride, Peak 3: osthole. H. The three peaks were also observed in the liquid chromatogram of DHJSD extract at 230 nm.


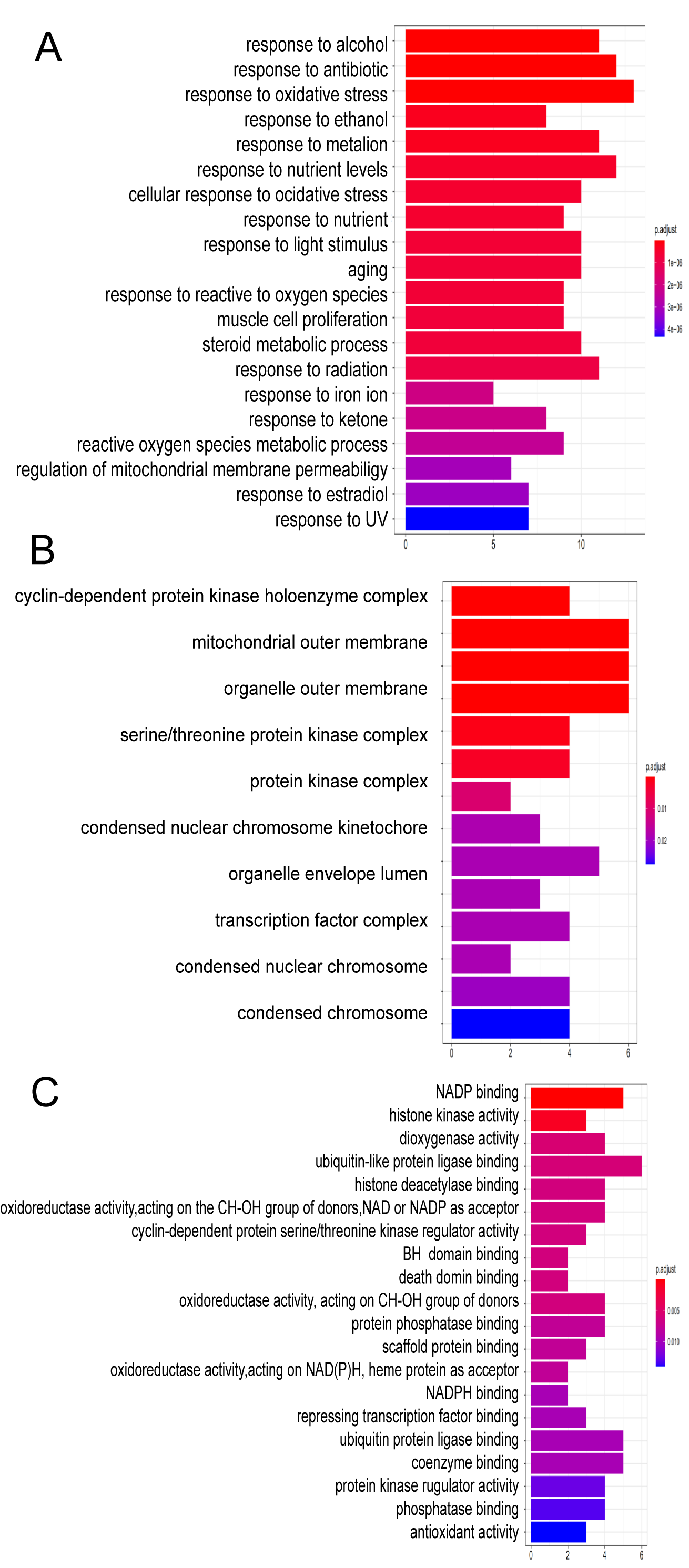


**Supplementary Figure 2.** Gene ontology (GO) terms for the candidate targets of DHJSD activity against OA for series 1. A.BP (biological process), B. CC (cellular component), C. MF (molecular function).


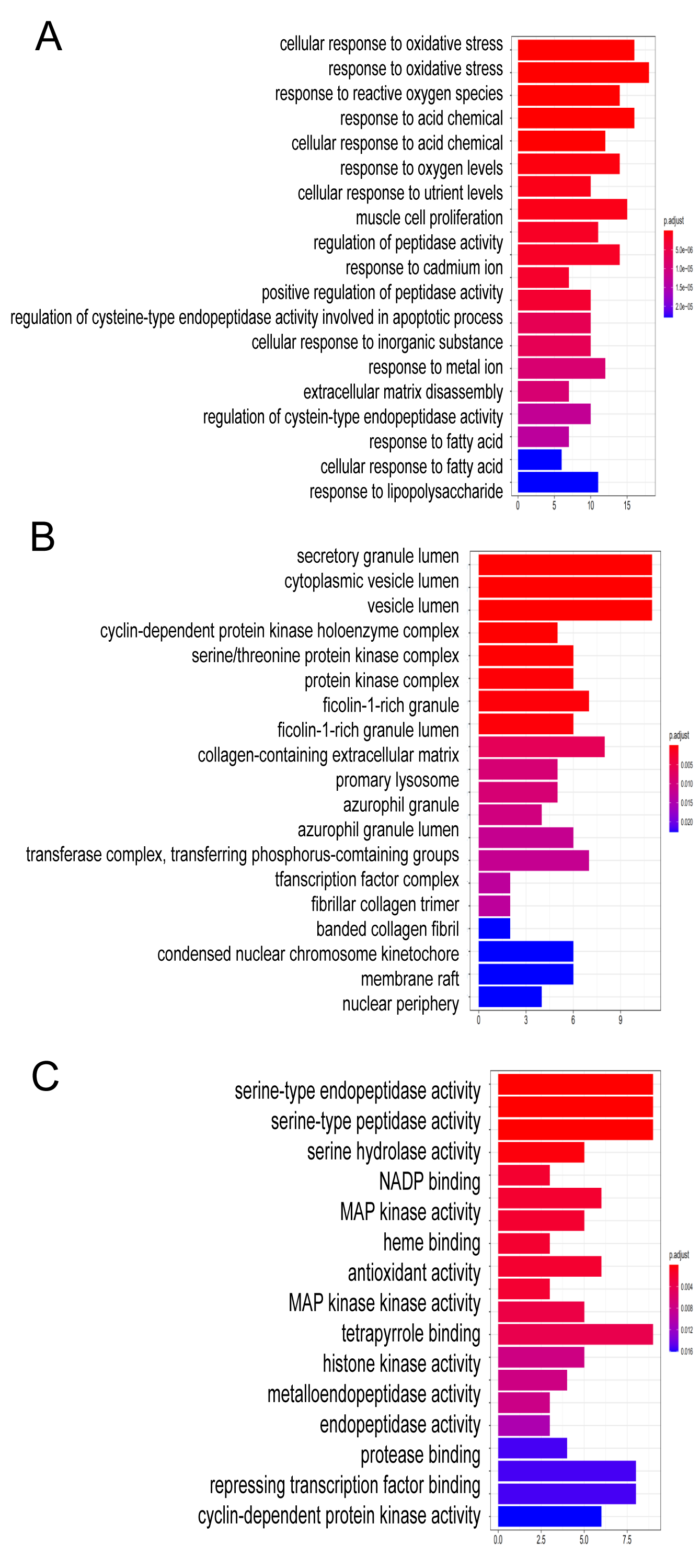


**Supplementary Figure 3.** Gene ontology (GO) terms for the candidate targets of DHJSD activity against OA for series 2. A.BP (biological process), B. CC (cellular component), C. MF (molecular function).


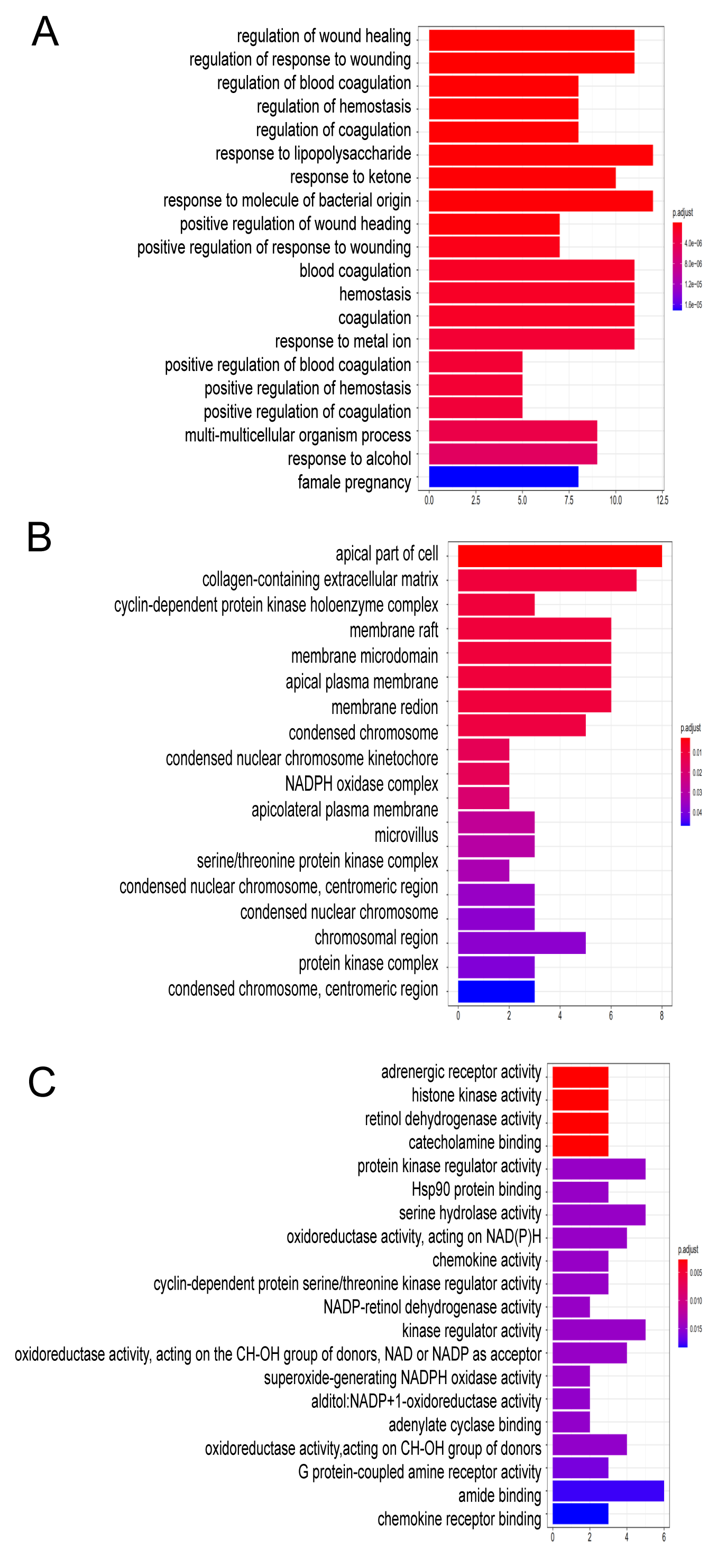


**Supplementary Figure 4.** Gene ontology (GO) terms for the candidate targets of DHJSD activity against OA for series 3. A.BP (biological process), B. CC (cellular component), C. MF (molecular function).

**
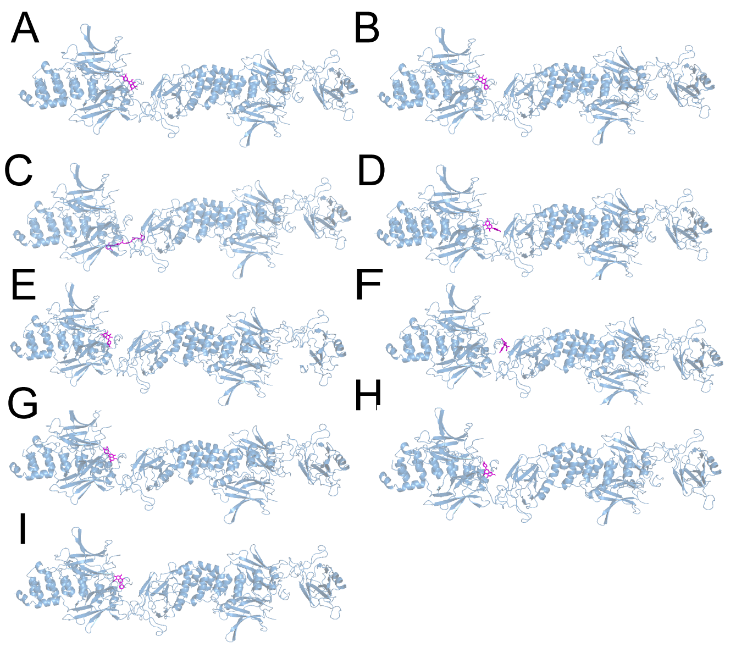
Supplementary Figure 5.** The docking between the top nine compounds［quercetin (A), baicalein (B), beta-carotene (C), isorhamnetin (D), kaempferol (E), licochalcone a(F), luteolin (G), naringenin (H) and wogonin (I)］of Duhuo Jisheng Decoction(DHJSD) and Osteoarthritis target RELA. The crystal structures of RELA was shown in blue and the compound was shown in purple.

**
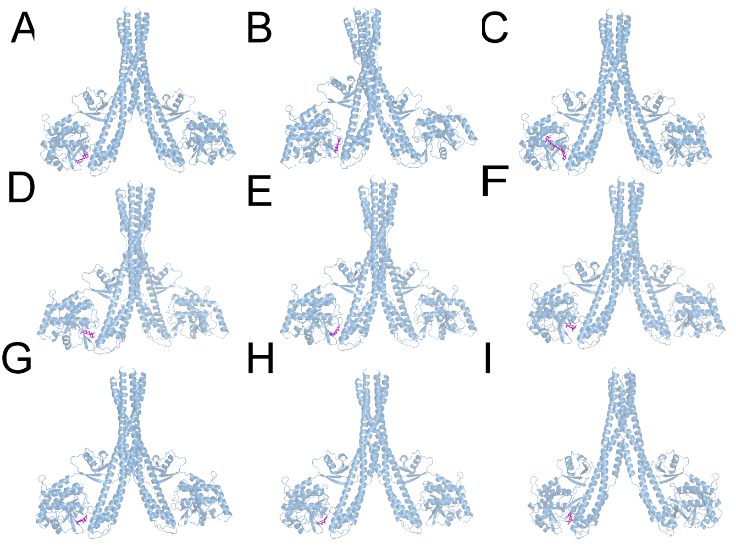
**

**Supplementary Figure 6.** The docking between the top nine compounds［quercetin (A), baicalein (B), beta-carotene (C), isorhamnetin (D), kaempferol (E), licochalcone a(F), luteolin (G), naringenin (H) and wogonin (I)］of Duhuo Jisheng Decoction(DHJSD) and Osteoarthritis target CHUK. The crystal structures of CHUK was shown in blue and the compound was shown in purple.

**Table S1. Ingredients of Duhuo Jisheng Decoction**

| **Chinese name** | **Pinyin**  **(Chinese phonetic alphabet）** | **English name** | **Scientific name** | **Part used** | **Proportion of ingredients (100%)** |
| --- | --- | --- | --- | --- | --- |
| 独 活 | Du Huo (DH) | Radix Angelicae Pubescentis | Angelica biserrata(Shan et Yuan) Yuan et Shan | Dried Root | 10.000% |
| 桑寄生 | Sang Ji Sheng (SJS) | Herba Taxilli | Taxillus chinensis (DC.) Danser | Dried Leaf stem branches | 6.667% |
| 秦 艽 | Qin Jiao (QJ) | Radix Gentianae Macrophyllae | Gentiana Macrophylla Pall. | Dried Root | 6.667% |
| 防 风 | Fang Feng (FF) | Radix Saposhnikoviae | Saposhnikovia divaricata (Turcz.) Schischk. | Dried Root | 6.667% |
| 细 辛 | Xi Xin (XX) | Herba Asari | Manchurian Wildginger | Dried Root and rhizome | 3.333% |
| 川 芎 | Chuan Xiong (CX) | Rhizoma Ligustici Chuanxiong | Szechuan Lovage Rhizome | Dried Root and rhizome | 6.667% |
| 当 归 | Dang Gui （DG） | Radix Angelicae Sinensis | Angelica sinensis (Oliv.) Diels. | Dried Root | 6.667% |
| 熟地黄 | Shu Di Huang (SDH) | Radix Rehmanniae Preparata | Rehmanniaglutinosa (Gaertn.) DC. | Prepared Root | 6.667% |
| 白 芍 | Bai Shao (BS) | Radix Paeoniae Alba | Paeonia lactiflora Pall | Dried Root | 6.667% |
| 肉 桂 | Rou Gui (RG) | Cortex Cinnamomi | Cortex Cinnamomi Cassiae | Dried Bark | 6.667% |
| 茯 苓 | Fu Ling (FL) | Poria | Smilax glabra Roxb | Dried Sclerotium | 6.667% |
| 杜 仲 | Du Zhong (DZ) | Cortex Eucommiae | Eucommia ulmoides Oliv | Dried Bark | 6.667% |
| 牛 膝 | Niu Xi (NX) | Radix Achyranthis Bidentatae | Radix Achyranthis Bidentatae | Dried Root | 6.667% |
| 党 参 | Dang Shen (DS) | Radix Ginseng | Root of Pilose Asiabell | Dried Root | 6.667% |
| 甘 草 | Gan Cao (GC) | Radix Glycyrrhizae | Glycyrrhiza uralensis Fisch. | Dried Root and rhizome | 6.667% |

**Table S2. The binding free energy between the top nine compounds with RELA**

| **Compound** | **Molecular formula** | | **CAS** | **docking score (kcal/mol)** |
| --- | --- | --- | --- | --- |
| Quercetin | C_15_H_10_O_7_ | 117-39-5 | | −9.1 |
| Baicalein | C_15_H_10_O_5_ | 491-67-8 | | −8.6 |
| Beta-carotene | C_40_H_56_ | 7235-40-7 | | −8.1 |
| Isorhamnetin | C_16_H_12_O_7_ | 480-19-3 | | −8.5 |
| Kaempferol | C_15_H_10_O_6_ | 520-18-3 | | −9.1 |
| Licochalcone a | C_21_H_22_O_4_ | 58749-22-7 | | −7.2 |
| Luteolin | C_15_H_10_O_6_ | 491-70-3 | | −9.1 |
| Naringenin | C_15_H_12_O_5_ | 480-41-1 | | −8.0 |
| Wogonin | C_16_H_12_O_5_ | 632-85-9 | | −7.9 |

**Table S3. The binding free energy between the top nine compounds with CHUK**

| **Compound** | **Molecular formula** | | **CAS** | **docking score (kcal/mol)** |
| --- | --- | --- | --- | --- |
| Quercetin | C_15_H_10_O_7_ | 117-39-5 | | −8.6 |
| Baicalein | C_15_H_10_O_5_ | 491-67-8 | | −8.7 |
| Beta-carotene | C_40_H_56_ | 7235-40-7 | | −8.1 |
| Isorhamnetin | C_16_H_12_O_7_ | 480-19-3 | | −7.7 |
| Kaempferol | C_15_H_10_O_6_ | 520-18-3 | | −8.4 |
| Licochalcone a | C_21_H_22_O_4_ | 58749-22-7 | | −7.8 |
| Luteolin | C_15_H_10_O_6_ | 491-70-3 | | −8.8 |
| Naringenin | C_15_H_12_O_5_ | 480-41-1 | | −8.6 |
| Wogonin | C_16_H_12_O_5_ | 632-85-9 | | −8.4 |
